# Variable selection for binary classification using error rate *p*-values applied to metabolomics data

**DOI:** 10.1186/s12859-015-0867-7

**Published:** 2016-01-14

**Authors:** Mari van Reenen, Carolus J. Reinecke, Johan A. Westerhuis, J. Hendrik Venter

**Affiliations:** Biosystems Data Analysis, Swammerdam Institute for Life Sciences, University of Amsterdam, Science Park 904, 1098 XH Amsterdam, The Netherlands; Centre for Business Mathematics and Informatics, Faculty of Natural Sciences, North-West University (Potchefstroom Campus), Private Bag X6001, Potchefstroom, South Africa; Centre for Human Metabolomics, Faculty of Natural Sciences, North-West University (Potchefstroom Campus), Private Bag X6001, Potchefstroom, South Africa; Department of Statistics, Faculty of Natural Sciences, North-West University (Potchefstroom Campus), Private Bag X6001, Potchefstroom, South Africa

**Keywords:** Variable selection, Significance testing, Non-parametric, Binary classification, Metabolomics

## Abstract

**Background:**

Metabolomics datasets are often high-dimensional though only a limited number of variables are expected to be informative given a specific research question. The important task of selecting informative variables can therefore become complex. In this paper we look at discriminating between two groups. Two tasks need to be performed: (i) finding variables which differ between the two groups; and (ii) determining how the selected variables can be used to classify new subjects. We introduce an approach using minimum classification error rates as test statistics to find discriminatory and therefore informative variables. The thresholds resulting in the minimum error rates can be used to classify new subjects. This approach transforms error rates into *p*-values and is referred to as ERp.

**Results:**

We show that non-parametric hypothesis testing, based on minimum classification error rates as test statistics, can find statistically significantly shifted variables. The discriminatory ability of variables becomes more apparent when error rates are evaluated based on their corresponding *p*-values, as relatively high error rates can still be statistically significant. ERp can handle unequal and small group sizes, as well as account for the cost of misclassification. ERp retains (if known) or reveals (if unknown) the shift direction, aiding in biological interpretation. The threshold resulting in the minimum error rate can immediately be used to classify new subjects.

We use NMR generated metabolomics data to illustrate how ERp is able to discriminate subjects diagnosed with *Mycobacterium tuberculosis* infected meningitis from a control group. The list of discriminatory variables produced by ERp contains all biologically relevant variables with appropriate shift directions discussed in the original paper from which this data is taken.

**Conclusions:**

ERp performs variable selection and classification, is non-parametric and aids biological interpretation while handling unequal group sizes and misclassification costs. All this is achieved by a single approach which is easy to perform and interpret. ERp has the potential to address many other characteristics of metabolomics data. Future research aims to extend ERp to account for a large proportion of observations below the detection limit, as well as expand on interactions between variables.

**Electronic supplementary material:**

The online version of this article (doi:10.1186/s12859-015-0867-7) contains supplementary material, which is available to authorized users.

## Background

A major aim of metabolomics studies is to find metabolites that distinguish a control group of reference or “normal” subjects from a group of experimental or “abnormal” subjects which differ from the control group subjects as a result of disease, treatment with drugs, toxicity, environmental, genetic or physiological effects [1–3]. The interpretation of those metabolites in terms of the underlying biological phenomena and the development of discriminating biomarkers are important goals [4]. Traditional statistical methods often make assumptions which make the validity of results questionable in the case of metabolomics. Metabolite concentrations are non-negative, requiring suitable transformation to accommodate the distributional assumptions of parametric statistical methods. While non-parametric methods such as the Mann–Whitney test make no distributional assumptions, they do not produce classification rules for new subjects. Metabolomics datasets generated through spectroscopic or spectrometric methods can consist of hundreds and even thousands of variables, making the selection of discriminatory variables an important yet complex task [5]. In addition, it is often difficult or expensive to obtain sufficient subjects or sample material to make group sizes large and equal. However, methods such as logistic regression require large sample sizes (*N*), especially when confronted with a large number of explanatory variables (*V*) [6]. Variable selection prior to developing a classification model is often performed on large *V* small *N* data. However, inference after variable selection is not advisable without correcting for the uncertainty associated with the selection step [7]. These methods also require model specifications such as linearity in the variables of the regression function. This linearity may well be misspecified. Methods such as PLS-DA (Partial least squares regression for discriminant analysis) are often used, but are biased and more likely to classify new subjects as belonging to the smaller of the two groups [8], whereas the cost of misclassification may be opposite to this. Furthermore, variable selection based on a PLS-DA model is still problematic [9]. Such projection based statistical methods are generally rather sophisticated, limiting their practicality [4, 8]. This is likely the reason why metabolomics researchers often combine the results of a variety of statistical and even machine learning methods to select a subset of variables. Doing so can become cumbersome if they do not reach the same decision and again requires estimation of “post-selection error” [10] when used in further model development.

The notions of sensitivity (*se*) and specificity (*sp*) of statistical methods are often used to evaluate the classification ability of models. These can be combined into the Youden Index (*J*) and the area under the receiver operating characteristic curve (AUC) [11, 12]. In this paper we also combine sensitivity and specificity but in the form of a weighted sum of misclassification error rates depending on a threshold. The choice of threshold resulting in the minimum error rate provides us with a rule to classify new subjects. There are some parallels with CART (classification and regression trees) in this respect. However, we show that non-parametric hypothesis testing can be based on the minimised error rates. This enables us to convert the error rates into *p*-values which in turn lead to selecting variables that contain discriminatory information. These *p*-values provide a ranking of the variables and the notion of multiple testing corrections can be used to decide how far up in this ranking the variables are considered to contain significant discriminatory information. This approach is referred to as ERp below.

ERp takes unequal and small group sizes explicitly into account and allows for the specification of the relative cost of misclassification, which is desirable when selecting an appropriate threshold. ERp provides information regarding the direction of the shift, i.e., whether metabolite levels are higher or lower in the experimental group, thus aiding biological interpretation of the results. ERp simultaneously provides us with a classification rule which can be applied immediately to classify new subjects. That is, once statistically significantly shifted variables have been selected, final classification can be based on a majority vote (as used here) or a more complex weighting structure taking *p*-values into account.

The paper is structured as follows: First, we review the notion of classification error rates and their dependence on thresholds, as well as how they can be estimated from available data. Secondly, we show that the testing used in ERp is non-parametric and how to calculate the relevant *p*-values. Finally, we illustrate the application of ERp on ^1^H nuclear magnetic resonance (NMR) spectroscopy data from cerebrospinal fluid (CSF) samples to discriminate subjects diagnosed with *Mycobacterium tuberculosis* infected meningitis from a control group and compare our results with those obtained using traditional methods [13].

## Methods

### Introduction

If there is a shift in the concentration of a metabolite from the control to the experimental group, the shift is either upwards or downwards. For such a metabolite there is a concentration threshold which can be used to discriminate between the groups and classify new subjects. The combined error rate is associated with a choice of threshold and if it can be chosen to make this combined error rate small, the metabolite is important as a discriminator between the groups. This raises the question: How small must this error rate be for the associated variable (e.g., metabolite) to be a good discriminator? ERp makes use of significance testing and transformation of this error rate to a *p*-value to answer this question.

### Classification error rates

Consider a single variable *X* and let *F*_0_ and *F*_1_ denote the population cumulative distribution functions (CDFs) for control and experimental subjects respectively. We assume that experimental subjects tend to have either upwardly or downwardly shifted values of *X* when compared to control subjects. It is important to treat these shift directions separately in order to properly determine the role of *X* in discriminating between the groups.

The **upward rule** is to choose a threshold *c* and classify a subject as experimental group if *X > c* and as control if *X* ≤ *c*. The rate of misclassification of control subjects is 1 − *F*_0_(*c*) and of experimental subjects is *F*_1_(*c*). Let weights *w*_0_ and *w*_1_ (*w*_0_ + *w*_1_ = 1) represent the relative costs of misclassification of control and experimental subjects respectively, so that the weighted combined error rate is

1 $$ e{r}_{up}(c)={w}_0\left(1-{F}_0(c)\right)+{w}_1{F}_1(c). $$

Choosing equal weights ${w}_0={w}_1=\frac{1}{2}$ implies that it is equally important to keep both rates of misclassification low. In other applications the weights could be selected differently, e.g., if experimental subjects are ill individuals, then misclassifying an individual as an experimental subject may imply costly or invasive treatment, while not identifying an ill individual as an experimental subject may have fatal consequences. The incidence rates of the two groups may also need to be taken into account in the choice of the weights.

For the **downward rule** a subject is classified as experimental if *X* ≤ *c* and as control otherwise. The weighted combined error rate is then

2 $$ e{r}_{down}(c)={w}_0{F}_0(c)+{w}_1\left(1-{F}_1(c)\right). $$

Both error rates are functions of the threshold *c*. Let *c*_*up*_ and *c*_*down*_ represent the choices of *c* that minimize (1) and (2) with minimized error rates *er*_up_^*^ and *er*_down_^*^ respectively. If *er*_up_^*^ is small, the variable *X* can be used to classify subjects following the upward rule with threshold *c*_*up*_. Alternatively, if *er*_down_^*^ is small, the downward rule with threshold *c*_*down*_ can be applied.

As mentioned above a shift in distribution is either upward or downward and this is reflected in only one of the pair *er*_up_^*^ and *er*_down_^*^ being small. With this in mind, we introduce also a** minimum error rate** together with a threshold and direction by letting

3 $$ e{r}_{min}^{*}=e{r}_{up}^{*},\;{c}_{min}={c}_{up}\;\mathrm{and}\;{d}_{min}=" up"\;\mathrm{if}\;e{r}_{up}^{*}<e{r}_{down}^{*}\;\mathrm{and} $$

4 $$ e{r}_{min}^{*}=e{r}_{down}^{*},\;{c}_{min}={c}_{down}\;\mathrm{and}\;{d}_{min}=" down"\;\mathrm{if}\;e{r}_{up}^{*}\ge e{r}_{down}^{*} $$

In other words, *er*_min_^*^ is the minimum of the up and down error rates while *c*_*min*_ and *d*_*min*_ are the threshold and shift direction associated with this minimum. Sometimes subject matter reasons may dictate to only consider the upward or downward shift, but in the absence of such reasons, we choose the smaller of the two error rates (*er*_min_^*^). We can then classify a new subject using the rule specified by *d*_*min*_.

### Estimating error rates from data

All the quantities introduced above depend on the population CDFs *F*_0_ and *F*_1_ which are usually unknown and can only be estimated from the data at hand. Notation is needed for this purpose. Denote the number of subjects observed in the control and experimental groups by *N*_0_ and *N*_1_ respectively. Let *N* = *N*_0_ + *N*_1_, and for *n* = 1, 2, …, *N*, let *x*_*n*_ represent the value of *X* for the *n*-th subject and *y*_*n*_ its group indicator taking the value 0 for the control group and 1 for the experimental group.

The empirical estimates of *F*_0_(*c*) and *F*_1_(*c*) are given by $\frac{1}{N_0}{\displaystyle {\sum}_{n=1}^N\left(1-{y}_n\right)I\left({x}_n\le c\right)}$ and $\frac{1}{N_1}{\displaystyle {\sum}_{n=1}^N{y}_nI\left({x}_n\le c\right)}$ respectively, where *I*(*A*) is the indicator function of the event *A*. Replacing *F*_0_(*c*) and *F*_1_(*c*) in (1) by their estimates, the estimated combined error rate is

5 $$ {\widehat{er}}_{up}(c)=\frac{w_0}{N_0}{\displaystyle {\sum}_{n=1}^N\left(1-{y}_n\right)I\left({x}_n>c\right)+\frac{w_1}{N_1}{\displaystyle {\sum}_{n=1}^N{y}_nI\left({x}_n\le c\right)}} $$

Let *ĉ*_*up*_ denote the value of *c* which minimizes (5) and let the corresponding minimized error rate be ${\widehat{er}}_{up}\left({\widehat{c}}_{up}\right)=mi{n}_c\left\{{\widehat{er}}_{up}(c)\right\}={\widehat{er}}_{up}^{*}$.

This minimization can be performed by ranking the *x*_*n*_’s increasingly. As *c* is varied, ${\widehat{er}}_{up}(c)$ remains constant between the successively ranked *x*_*n*_ values. Hence it is sufficient to compute ${\widehat{er}}_{up}(c)$ only at the midpoints of the intervals formed by the ranked *x*_*n*_ values and then to choose *ĉ*_*up*_ as the value which minimizes ${\widehat{er}}_{up}(c)$ [14].

Thus *ĉ*_*up*_ and ${\widehat{er}}_{up}^{*}$ provide estimates of *c*_*up*_ and *er*_up_^*^ respectively when using the upward rule. If an upward shift in the values of the variable *X* is of interest and ${\widehat{er}}_{up}^{*}$ turns out to be small, *X* can be used to classify subjects by applying the upward rule with threshold *ĉ*_*up*_. Similar statements hold when specifying a downward shift or specifying no shift direction (see Additional file 1: Figure S1).

### Using classification error rates as test statistics

Clearly, the discriminating ability of *X* is related to the size of ${\widehat{er}}_{up}^{*}$, but it is not obvious how small ${\widehat{er}}_{up}^{*}$ should be for *X* to be a good discriminator. Furthermore, the true but unknown error rate *er*_up_^*^ may differ from ${\widehat{er}}_{up}^{*}$, making it inadvisable to judge the importance of the variable *X* solely on the value of ${\widehat{er}}_{up}^{*}$ without taking into account the extent of such differences. One possible way to do this is to calculate a standard error or confidence interval for ${\widehat{er}}_{up}^{*}$ as done when using the Youden index [12]. We propose to use ${\widehat{er}}_{up}^{*}$ as a test statistic to formally test the null hypothesis that there is no shift in the distribution of *X* for the experimental group compared to the control group, against the alternative of an upward shift in distribution. This enables us to judge the discriminatory importance of *X* in terms of the familiar concept of a *p*-value.

For testing purposes, the distributions of ${\widehat{er}}_{up}^{*},\;{\widehat{er}}_{down}^{*}\;\mathrm{and}\;{\widehat{er}}_{min}^{*}$ under the null hypothesis *H*_0_ : *F*(*x*) = *F*_0_(*x*) = *F*_1_(*x*) are required. Assume: (i) *F*(*x*) is a continuous and strictly increasing function in *x* starting at 0 for some sufficiently small value of *x*; and (ii) the *x*_*n*_’s are independent and identically distributed (*iid*) according to *F*. Putting *u*_*n*_ = *F*(*x*_*n*_) and *b* = *F*(*c*) equation (5) becomes

$$ \begin{array}{l}{\widehat{er}}_{up}(c)=\frac{w_0}{N_0}{\displaystyle {\sum}_{n=1}^N\left(1-{y}_n\right)I\left(F\left({x}_n\right)>F(c)\right)+\frac{w_1}{N_1}{\displaystyle {\sum}_{n=1}^N{y}_nI\left(F\left({x}_n\right)\le F(c)\right)}}\\ {}\kern1.92em =\frac{w_0}{N_0}{\displaystyle {\sum}_{n=1}^N\left(1-{y}_n\right)I\left({u}_n>b\right)+\frac{w_1}{N_1}{\displaystyle {\sum}_{n=1}^N{y}_nI\left({u}_n\le b\right)}}\\ {}\kern1.92em ={\tilde{er}}_{up}(b)\end{array} $$

Since this expression is only a function of *b*, minimising over *c* is equivalent to minimising over *b*, giving ${\widehat{er}}_{up}^{*}=mi{n}_c\left\{{\widehat{er}}_{up}(c)\right\}=mi{n}_b\left\{{\tilde{er}}_{up}(b)\right\}$. This expresses ${\widehat{er}}_{up}^{*}$ as a function of the *u*_*n*_’s only. The probability integral transform states that the *u*_*n*_’s are independent and identically uniformly distributed on [0, 1], henceforth abbreviated as *IIUD *[0, 1]. Therefore, the null distribution of ${\widehat{er}}_{up}^{*}$ does not depend on *F*, i.e., ${\widehat{er}}_{up}^{*}$ is a non-parametric test statistic. Additional file 1: Figure S2 shows that this is also true for ${\widehat{er}}_{down}^{*}$ and ${\widehat{er}}_{min}^{*}$ and that the null distribution of ${\widehat{er}}_{down}^{*}$ is the same as for ${\widehat{er}}_{up}^{*}$. Moreover, the null distributions depend on the group sizes (*N*_0_ and *N*_1_) and the weights (*w*_0_ and *w*_1_), so that one does not need to be concerned about unequal sample sizes, sampling variability and biases when judging the resulting *p*-values as indicators of the discriminatory importance of *X* (see Additional file 1: Figure S4).

The actual calculation of the null distribution by analytic means seems impossible in view of the complicated expressions involved in the definitions of the error rates. However, simulation offers a solution. Table 1 provides an algorithm to convert error rate test statistics into their corresponding *p*-values. As an alternative to simulating the null distribution, asymptotic approximation can be used, as discussed in Additional file 1: Figure S3. We illustrate the benefit of converting error rates into their corresponding *p*-values in the Results & Discussion section below.

**Table 1:** Algorithm to simulate the null cumulative distribution functions

• Generate *N IIUD*[0,1] *u*_*n*_’s
• Assign the first *N*_0_ *y*_*n*_’s as 0 and the remainder as 1
• Minimize $\frac{w_0}{N_0}{\displaystyle {\sum}_n^N\left(1-{y}_n\right)I\left({u}_n>b\right)+\frac{w_1}{N_1}{\displaystyle {\sum}_{n=1}^N{y}_nI\left({u}_n\le b\right)}}$ by varying b over the midpoints of the increasingly ordered *u*_*n*_’s to obtain ${\widehat{er}}_{up}^{*}$
• Repeat these steps *M* times to build up a file of *iid* copies of ${\widehat{er}}_{up}^{*}$, say ${\widehat{er}}_{up}^{*}(m),\;m=1,\dots, M$, whose empirical distribution function provides a simulation approximation of the null CDF
• If *T* of the ${\widehat{er}}_{up}^{*}(m)\hbox{'}s$ fall below an actually observed ${\widehat{er}}_{up}^{*}$ its associated *p*-value is approximately *T*/*M*. Approximations are more accurate for large *M*.

### ERp applied to more than one variable

Up to this point we have considered only one variable *X*. As mentioned previously, metabolomics studies deal with multiple variables, therefore we need to find a short list of discriminatory and biologically relevant variables which are preferably easy to detect in clinical practice [4]. ERp calculates the error rate for each variable, converts it into a *p*-values and ranks the variables by increasing *p*-values. The family wise error rate (FWER) or false discovery rate can be used to decide how far up the ranking variables are still considered to contain significant information. We use the Bonferroni-Holm (BH) method [15] to control the FWER for multiple testing, making it the only parameter involved in our approach. However, a slight adjustment is required when applying stepwise methods such as BH, since more than one variable can have the same error rate and therefore *p*-value. The stepwise nature of BH may indicate that some variables are significant while others, with the same error rate, are not. In such instances we prefer to be on the conservative side regarding control of the FWER. That is, if a variable is not significant then all variables with the same error rate should also be treated as not significant. Instead of using the Bonferroni-Holm method the user may opt for any of the many other available correction methods (see for example [16] and [17]).

### ERp software

The Matlab [18] functions to perform ERp, as well as an example application, are provided as part of the Additional file 2. Additional file 1: Figure S8 gives details of this software together with the user inputs required and the output delivered.

## Results and discussion

In this section we discuss two examples to illustrate the benefit of converting error rates into their corresponding *p*-value. The sample sizes *N*_0_ = 21 and *N*_1_ = 12 were selected to correspond with those of the dataset used to illustrate ERp in the application subsection below. Two weight sets, referred to as 1 $\left({w}_0={w}_1=\frac{1}{2}\right)\;\mathrm{and}\;2\;\left({w}_0=\frac{1}{3}\;\mathrm{and}\;{w}_1=\frac{2}{3}\right)$ are used. These examples are used throughout the remainder of the paper.

### Converting error rates to *p*-values

**Fig. 1:**
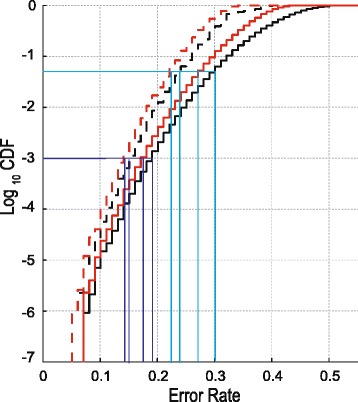
The null cumulative distribution functions. The graphs show the *log*_10_ transformed null CDFs of ${\widehat{er}}_{up}^{*}$ (black lines) and ${\widehat{er}}_{min}^{*}$ (red line), for group sizes *N*_0_ = 21 and *N*_1_ = 12 using weight sets 1 (solid lines) and 2 (dashed lines). The dark (α = 0.001) and light (α = 0.05) blue lines represent points of reference discussed in the text

We calculate the null CDFs based on one million simulation repetitions using the algorithm in Table 1. Since we are only interested in significantly small error rates, the left tails of the null distributions are relevant. For clarity purposes, Fig. 1 shows the left tails of the logarithms of these null CDFs. As is to be expected the null distribution of ${\widehat{er}}_{min}^{*}$ is shifted to the left relative to that of ${\widehat{er}}_{up}^{*}$. This is because ${\widehat{er}}_{min}^{*}$ has slightly less power being a two-sided test compared to the one-sided test of ${\widehat{er}}_{up}^{*}$. The customary 5 % significance level (*α* = 0.05 = 10^− 1.3^) is attained for error rates as high as 0.3 and 0.25 (light blue lines). An error rate as large as 0.3 or 0.25 would likely not lead one to conclude that *X* has discriminatory ability. This ability becomes more apparent when evaluating the corresponding *p*-values. Metabolomics studies have many variables requiring correction for multiple testing. Therefore, a lower significance level such as *α* = 0.001 = 10^− 3^ may be relevant. Even for such a low α the observed error rates are above 0.15 (dark blue lines). Additional file 1: Figure S4 discusses similar results for other sample sizes and weight combinations.

### Power comparison of error rate test statistics

ERp operates on two levels, namely it performs a hypothesis test and also delivers a classification threshold. It may be anticipated that delivering two outputs comes at a cost, i.e., less power in the hypothesis testing part. Here we briefly report a simulation study comparing the power of ${\widehat{er}}_{up}^{*}$ and ${\widehat{er}}_{min}^{*}$ as test statistics against that of the well-known non-parametric Mann–Whitney (MW) test. The results presented here assume that the control group follows a standard log-normal distribution, while the experimental group follows an upwardly shifted log-normal distribution. That is, if *y*_*n*_ = 0, then *X*_*n*_ = exp(*Z*_*n*_) with *Z*_*n*_ normally distributed with mean 0 and variance 1, while if *y*_*n*_ = 1, then *X*_*n*_ = exp(*Z*_*n*_ + *μ*) where *μ* varies over a grid of positive values. Ten thousand simulated data sets were generated for each grid point and the *p*-values were calculated for the test statistics ${\widehat{er}}_{up}^{*},\;{\widehat{er}}_{min}^{*}$ and MW, at each shift magnitude and for each repetition. These simulations were performed in Matlab [18] making use of the one-sided MW test ensuring sound comparison to ${\widehat{er}}_{up}^{*}$.

**Fig. 2:**
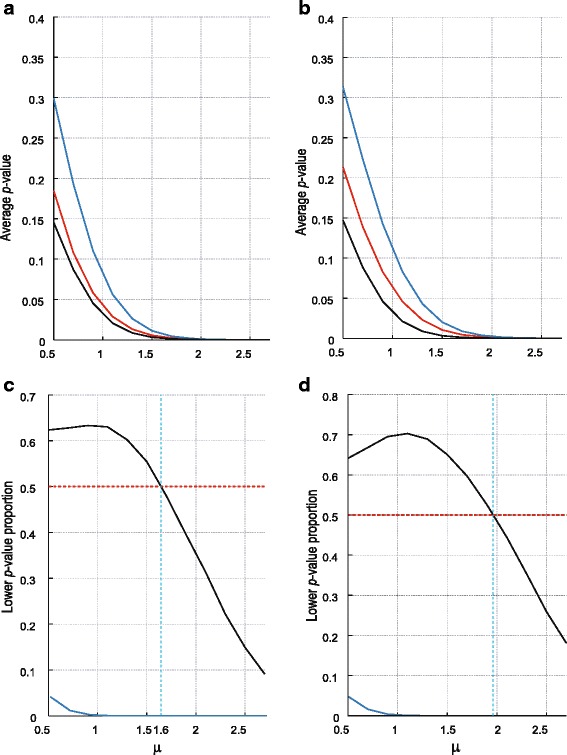
Simulation comparison of the different error rate test statistics. Figures **a** (weight set 1) and **b** (weight set 2) depict the average *p*-values associated with ${\widehat{er}}_{up}^{*}$ (red lines), ${\widehat{er}}_{min}^{*}$ (blue lines) and the MW test statistic (black lines). Figures **c** (weight set 1) and **d** (weight set 2) depict the proportions of repetitions in which the *p*-values of ${\widehat{er}}_{min}^{*}$ (blue lines) and MW (black lines) were below the *p*-values of ${\widehat{er}}_{up}^{*}$. The dotted red line represents the 50 % cut-off. The dashed blue lines represent points of reference as discussed in the text

As a first measure of comparison the resulting *p*-values were averaged over the repetitions to measure the expected power of the test statistics. Figure 2a and b represent graphs for weight set 1 and 2, respectively. As is to be expected, having a priori information regarding which shift direction to evaluate does improve the discriminatory ability of ERp, since ${\widehat{er}}_{up}^{*}$ is more powerful than ${\widehat{er}}_{min}^{*}$. It is also evident that the MW test statistic delivers more power on average, but this diminishes at larger shift, i.e., at shifts of most practical relevance.

As a second measure of comparison, Fig. 2c and d show the proportion of *p*-values for each of the test statistics that are smaller than the *p*-values of ${\widehat{er}}_{up}^{*}$. If this proportion is above (below) 50 %, then the corresponding test is frequently better (poorer) than ${\widehat{er}}_{up}^{*}$ on this measure. These figures show that the MW is better at smaller shifts, i.e., below 1.6 and 2.0, while for larger shifts, ERp is consistently better.

In summary, ERp’s ability to also classify new subjects does not seem to result in a lack of power as compared to the MW test. Additional file 1: Figure S6 supplies more results on this matter.

### Leave-one-out error rate estimation

In the classification literature leave-one-out (LOO) cross-validation is often used to estimate error rates with less bias than the error rates used throughout this paper [19]. This leads to the question whether LOO error rates can also be used in a hypothesis testing role to find discriminating variables analogous to the approach presented above. However, lower bias of the LOO error rates does not automatically imply greater power in the testing context. We studied this issue and found that the LOO error rate based test statistics are also non-parametric but on average less powerful. Additional file 1: Figures S5 and S6 provide more details.

### Application to metabolomics data

Finally, we illustrate ERp by applying it to data obtained from the metabolomics study reported in Mason et al. [13]. The data was generated through ^1^H NMR spectroscopy from CSF samples obtained from subjects who suffered from meningitis, but not caused by *Mycobacterium tuberculosis* (Mtb) infection. They formed the control group. The experimental group consisted of patients who like-wise suffered from with meningitis, however confirmed to be caused by Mtb.

Mason et al. [13] selected 12 variables in the CSF that were able to distinguish the control from the experimental subjects. These variables were selected based on PLS-DA VIP values as well as univariate statistics. Quantitatively, two metabolites that yielded the greatest measures of importance (i.e., those most responsible for the separation) were highly elevated lactate and decreased glucose in the TBM subjects relative to values observed for the controls. These two metabolites indicate the well-known disturbances in the energy metabolism of several neurological disorders [20]. Further selected variables also support the energy perturbation caused by the infection of the meninges by the tuberculosis bacterium.

We apply ERp to the same data excluding subjects identified as outliers in Mason et al. [13] and including only identified metabolites, thus leaving us with 55 metabolites, 17 experimental and 30 control subjects. Next we split the dataset into two parts: a training set and a test set. The test set includes about 30 % of subjects from each group, randomly selected, and are not used to find important metabolites. The test set contains 5 experimental and 9 control subjects and is used to show the classification ability of ERp given new subjects. The training set therefore contains *N*_0_ = 21 control subjects while the experimental group consists of *N*_1_ = 12 subjects. Mason et al. [13] made no weight assumptions so that using weight set 1 (our equal weights scenario) is likely to yield more comparable results. However, weight set 2 may be more appropriate in this application since untreated TBM is mostly fatal [21]. Therefore, results are reported for both weight sets. Although in this context the shift directions are known for some variables we elect to make no directional assumptions so as to allow the data to speak in this regard.

#### List of significantly shifted variables

Table 2 lists the variables selected by ERp for different choices of the FWER α together with their error rates $\left(\mathrm{E}\mathrm{R}={\widehat{er}}_{min}^{*}\right)$ and position in the NMR spectrum, thresholds $\left(C={\widehat{c}}_{min}\right)$, shift directions, *p*-values and the BH adjusted critical levels for the corresponding FWER. To illustrate how sensitive the list of selected variables is to the specified FWER, three choices are reported namely α = 1, 5 and 10 %. As is to be expected, a more tolerant FWER results in a longer list of significant variables.

**Table 2:**
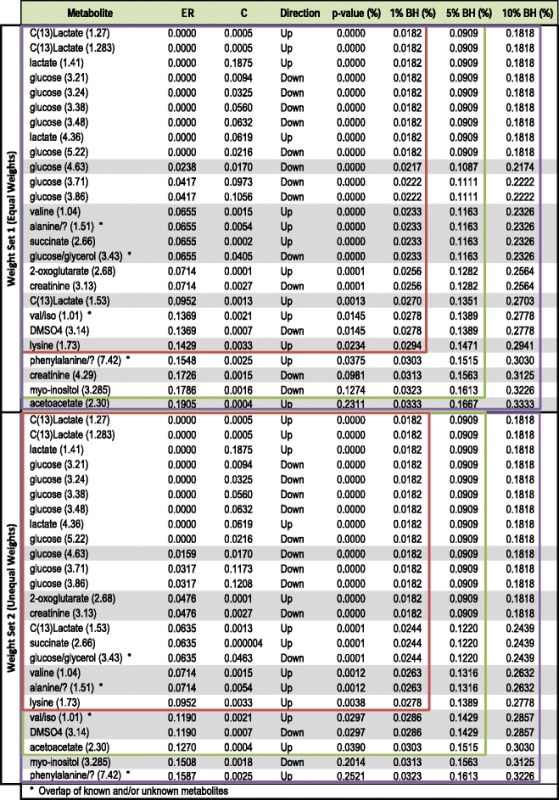
Significant variables based on weight set 1 and 2

The first column provides the variable names ordered according to increasing error rates which are shown in the second column. The third column provides the threshold estimates which can be used to classify new subjects by employing the “up” or “down” rule as indicated by the direction in the fourth column. The fifth column provides the *p*-values associated with the error rates, expressed as percentages. The significance of these values can be determined through comparison to the BH adjusted critical level. The last three columns provide these levels for three different FWERs namely 1, 5 and 10 %. The red, green and purple blocks encapsulate the variables that were significantly shifted at a 1, 5 and 10 % FWERs, respectively. For groups of variables with the same error rates and therefore the same *p*-values the most conservative BH level is applied. These groups are indicated in alternating blocks of white and grey

In addition to selecting the same metabolites as Mason et al. [13], ERp also selected succinate as a significant metabolite. Since succinate is an intermediate in the tricarboxylic acid cycle, the finding is compatible with the increased aerobic energy metabolism required by the microglia to destroy the invading tuberculosis bacterium. Overall, we are able to draw the same conclusions presented in [13] by using only one method as opposed to many different and more complex techniques.

**Fig. 3:**
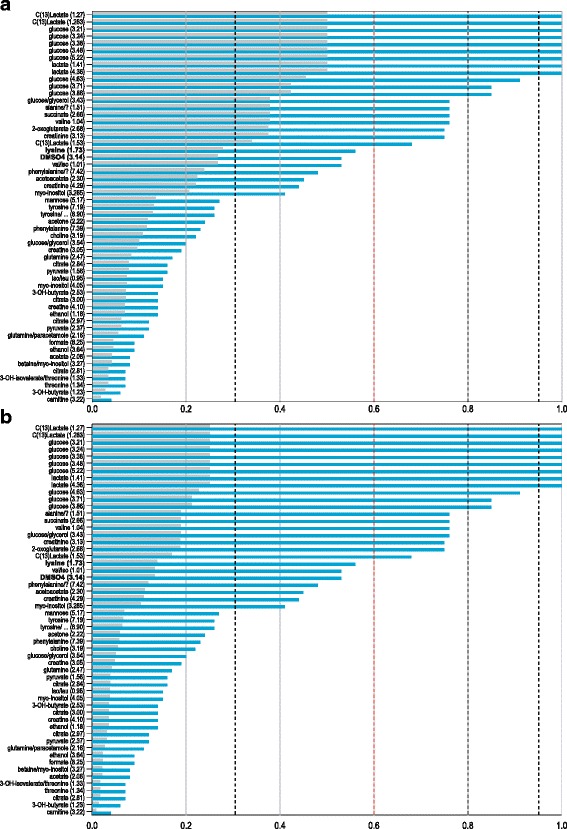
CART variable importance. The CART method provides a measure of importance for each variable. These values (grey bars), along with the normalized values (blue bars), are depicted here in the form of a bar chart for each variable (y-axis) in order of decreasing importance. Figures **a** and **b** represent weight sets 1 and 2 respectively. The vertical black and red dashed lines represent values at which large drops in Normalized Importance occur and therefore possible cut-off choices for variable selection. The dashed red lines represent the cut-offs chosen for comparison with ERp, while the dashed black lines represent alternative choices

For comparative purposes, we also modelled a standard CART classification tree, using SPSS [22], with the following specifications: (i) all variables were selected as potential predictors; (ii) equal prior probabilities for the two groups; (iii) stopping when the final nodes contain only one subject or at a tree depth of 100; and (iv) the test set was used for validation purposes. These settings were selected to make ERp and CART results as comparable as possible. CART constructs a classification tree by recursively dividing the data into subsets until these subsets are as homogeneous as possible with regard to group labels [23]. As a result, the tree stopped growing too soon and only included the variable “C13 lactic (1.27)”, overlooking other variables important for biological interpretation. CART does provide a measure of variable importance. Figure 3 displays these scores obtained when applying CART to the training data given the two weight sets. The suggested cut-off for variable selection corresponds to a point just before a large drop in Normalized Importance (NI). We chose a cut-off corresponding to an NI of 60 %, that is, all variables with NI exceeding 60 % were selected as important. At this cut-off CART did not flag two biologically important variables, compared to the original paper [13], and missed DMSO (the depletion of DMSO is associated with oxidative stress) and lysine (increased levels of lysine are associated with mental retardation). CART does not select variables with similar information to those already in the tree structure and may overlook metabolites necessary for biological interpretation.

#### Classification of unseen subjects

We now make use of the test set of subjects to illustrate the classification feature of ERp. Table 3 shows the classification results based on the lists of variables in Table 2, taking α = 1 %, using the corresponding thresholds, shift directions and classification rules.

**Table 3:**
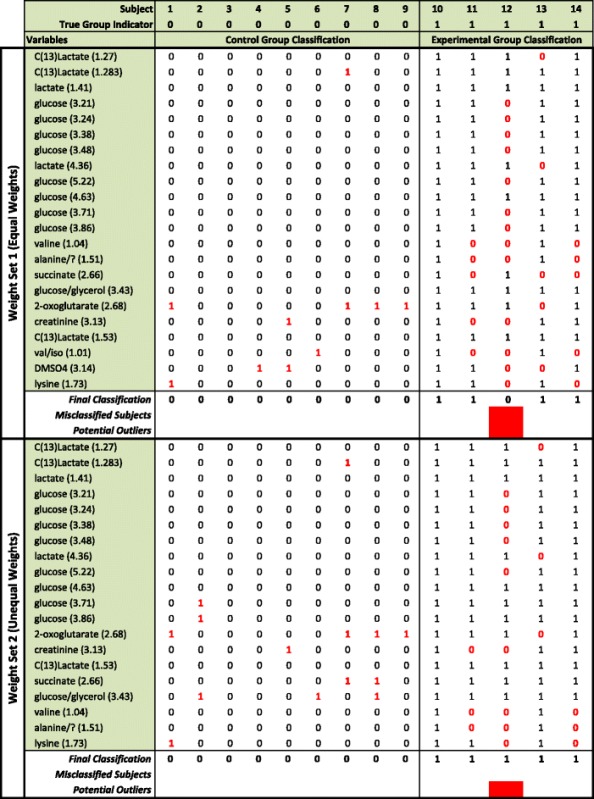
Group classification and outlier detection using significant variables based on weight set 1 and 2

The body of the table shows the classification result due to each significantly shifted variable for each subject, where 0 indicates the subject was classified into the control group and 1 indicates the subject was classified into the experimental group. Misclassifications are indicated in red. The last three rows (i) provide the final classification based on the majority vote; (ii) flag subjects that were misclassified; and (iii) flag potential outlying subjects based on the number of variables that misclassified it compared to the remaining subjects

The variables in Table 3 are ordered based on their *p*-value, from smallest to largest. As is to be expected variables lower down the list are prone to making more misclassifications. However, in general there are very few misclassifications. The majority of variables made a single misclassification and mostly for the same subject (number 12), indicating that it may be an “outlier”, i.e., not representative of the group. Constructing classification tables for test as well as training subjects enables us to screen for potential outliers, another potentially useful application of ERp. However, since outlier detection is not the main aim of this paper, we do not explore the matter further.

Overall the second weight set, which puts more weight on correctly classifying subjects in the experimental group, was more successful and made no final misclassifications, based simply on majority vote, even in the presence of the potential outlier. No classification model was developed in the original paper [13] and therefore no comparison is possible. The classification results for the test set (assuming equal weights) are the same for the CART model as for ERp, with only one experimental subject misclassified. However, ERp outperforms the CART model for weight set 2 (assuming unequal weights), with CART again misclassifying one experimental subject while ERp made no misclassifications.

Though this is only a single application and not a comprehensive comparison, the choice of cut-off for CART remains difficult to interpret. In contrast, ERp makes use of a controlled FWER (α) which has a direct interpretation as the probability of having included one or more variables which do not discriminate between the groups.

## Conclusion

Our main contribution has been to show that non-parametric hypothesis testing, based on minimum error rates, can find statistically significantly shifted variables. We found that the discriminatory ability of variables becomes more apparent when error rates were evaluated based on their corresponding *p*-values as relatively high error rates can still be significant. The power simulations performed concluded that the MW test is more powerful for small shifts in distribution, while ERp is competitive for larger shifts. An exploratory application of ERp indicated that markers of the disease state of patients suffering from TBM were successfully selected and used for the classification of patients with meningitis due to Mtb infection relative to other causes.

In summary, ERp can accommodate unequal and small group sizes while accounting for the cost of misclassification into either group. ERp retains (if known) or reveals (if unknown) the shift direction, aiding biological interpretation. The thresholds resulting in the minimum error rates can be used to classify new subjects or to identify potential outliers.

ERp is a useful addition to the range of methods used for binary discrimination and classification. Future research aims to explain how ERp can evaluate interactions and extend ERp to accommodate data with a large proportion of observations below the detection limit.

## Additional files

Additional file 1: **Supplementary Material.** (DOCX 2894 kb)

Additional file 2: **Matlab Code.** (ZIP 10 kb)
