# Supplementary material for: Variable selection for binary classification using error rate p-values applied to metabolomics data
Source: BMC Bioinformatics. 2016 Jan 14;17:33. doi: 10.1186/s12859-015-0867-7 (PMC4712617; doi:10.1186/s12859-015-0867-7)
Supplement: Supplementary file 1 — Supplementary Material. (DOCX 2894 kb) [file 12859_2015_867_MOESM1_ESM.docx]

**Supplementary Material: *Variable selection for binary classification using error rate*** $\boldsymbol{p}$***-values applied to metabolomics data***

**Introduction**

This document provides a summary of additional results that may be useful to the reader and should be read in conjunction with the paper. Note that only a discussion or summary is provided here for the sake of brevity, however the reader is encouraged to contact the corresponding author to obtain a comprehensive version of this document.

**Index**

| **Section** | | **Description** | | **Page number** | |
| --- | --- | --- | --- | --- | --- |
| S1 | | Estimating error rates from data | | 2 |  |
| S2 | | Using classification error rates as test statistics | | 3 |  |
| S3 | | Asymptotic estimation of the null distribution | | 4 |  |
| S4 | | Comparing null distributions | | 7 |  |
| S5 | | Tests based on leave-one-out error rates | | 9 |  |
| S6 | | Power comparisons of test statistics | | 11 |  |
| S7 | | Comparing threshold estimators | | 13 |  |
| S8 | | Summary of the ERp Approach | | 16 |  |

**Section S1:**

**Estimating error rates from data**

Replacing $F_{0}(c)$ and $F_{1}(c)$ in equation (2) in the main paper by their estimates, the estimated combined error rate is

$$\hat{er}_{down}\left( c \right)=\frac{w_{0}}{N_{0}}\sum_{n=1}^{N} (1-y_{n})I(x_{n}\leq c)+\frac{w_{1}}{N_{1}}\sum_{n=1}^{N} y_{n}I(x_{n}>c)$$

Denote by $\hat{c}_{down}$, the corresponding estimate of $c_{down}$, a choice of $c$ that minimises the equation above with minimized value $\hat{er}_{down}^{*}$. This minimization can be performed using the same approach as described in the main paper. These two items provide estimates of $c_{down}$ and ${er}_{down}^{*}$ based on the available data when using the downward rule. If an downward shift in the values of the variable $X$ is of interest and $\hat{er}_{down}^{*}$ turns out to be small, the variable $X$ can be used to classify subjects following the downward rule with threshold $\hat{c}_{down}$.

However, in the estimation context, we may not have a preference for either one of the two directions and allow the data to “speak” in this regard, i.e. to consider the smaller of the two directional error rates. Parallel to the notation in equations (3) and (4) in the main paper:

| $\hat{er}_{min}^{*}=\hat{er}_{up}^{*}$ , $\hat{c}_{min}=\hat{c}_{up}$ and $\hat{d}_{min}="up"$ if $\hat{er}_{up}^{*}<\hat{er}_{down}^{*}$ and |  |
| --- | --- |
| $\hat{er}_{min}^{*}=\hat{er}_{down}^{*}$ , $\hat{c}_{min}=\hat{c}_{down}$ and $\hat{d}_{min}="down"$ if $\hat{er}_{up}^{*}\geq\hat{er}_{down}^{*}$ |  |

represent the corresponding estimators of ${er}_{min}^{*}$, $c_{min}$ and $d_{min}$.

**Section S2:**

**Using classification error rates as test statistics**

Similarly to the proof provided in the paper for $\hat{er}_{up}\left( c \right)$, it follows that

| $\hat{er}_{down}\left( c \right)=\frac{w_{0}}{N_{0}}\sum_{n=1}^{N} (1-y_{n})I(u_{n}\leq b)+\frac{w_{1}}{N_{1}}\sum_{n=1}^{N} y_{n}I(u_{n}>b)$ $=\tilde{er}_{down}\left( b \right)$ |  |
| --- | --- |

Again $\hat{er}_{down}^{*}={min}_{c}\left\{ \hat{er}_{down}\left( c \right) \right\}={min}_{b}\left\{ \tilde{er}_{down}\left( b \right) \right\}$ which shows that $\hat{er}_{down}^{*}$ is also a non-parametric test statistic. Putting ${u_{n}^{'}=1-u}_{n}$ and $b^{'}=1-b$, $\hat{er}_{down}\left( c \right)$ can be expressed as

| $\hat{er}_{down}\left( c \right)=\frac{w_{0}}{N_{0}}\sum_{n=1}^{N} \left( 1-y_{n} \right)I\left( u_{n}^{'}\geq b^{'} \right)+\frac{w_{1}}{N_{1}}\sum_{n=1}^{N} y_{n}I\left( u_{n}^{'}<b^{'} \right)=\breve{er}_{down}\left( b^{'} \right)$ |
| --- |

so that $\hat{er}_{down}^{*}={min}_{c}\left\{ \hat{er}_{down}\left( c \right) \right\}={min}_{b^{'}}\left\{ \breve{er}_{down}\left( b^{'} \right) \right\}$.

The expressions for $\hat{er}_{down}^{*}$ and $\hat{er}_{up}^{*}$ are therefore the same except for possible equalities which carry zero probability and since the $u_{n}^{'}$’s have the same distribution as the $u_{n}$’s, it follows that $\hat{er}_{down}^{*}$ has the same distribution as $\hat{er}_{up}^{*}$.

Finally, under the null hypothesis $\hat{er}_{min}^{*}=min\left[ {min}_{b}\left\{ \tilde{er}_{up}\left( b \right) \right\},{min}_{b}\left\{ \tilde{er}_{down}\left( b \right) \right\} \right]$ which expresses $\hat{er}_{min}^{*}$ as a function of the $u_{n}$’s as well so that it too is a non-parametric test statistic.

**Section S3:**

**Asymptotic estimation of the null distribution**

A large sample approximation of the null distribution can be obtained by letting:

$F_{N_{0}}(b)=\frac{1}{N_{0}}\sum_{n=1}^{N} \left( 1-y_{n} \right)I\left( u_{n}\leq b \right)$ and $F_{N_{1}}(b)=\frac{1}{N_{1}}\sum_{n=1}^{N} y_{n}I(u_{n}\leq b)$

denote the respective empirical CDFs in the argument $b$ of the $u_{n}$’s corresponding to the control and experimental groups, then (9) may be written as:

$\tilde{er}_{up}\left( b \right)=w_{0}\left[ 1-F_{N_{0}}\left( b \right) \right]+w_{1}F_{N_{1}}\left( b \right)$.

It is well known that when sample sizes increase $\sqrt{N_{0}}\{F_{N_{0}}\left( b \right)-b\}$, taken as a stochastic process in $b$, tends in distribution to a standard Brownian bridge (BB) process and the same holds for $\sqrt{N_{1}}\{F_{N_{1}}\left( b \right)-b\}$[1,2]. Now:

$\tilde{er}_{up}\left( b \right)=w_{0}+{b(w}_{1}-w_{0})+\frac{w_{1}}{\sqrt{N_{1}}}\sqrt{N_{1}}\{F_{N_{1}}\left( b \right)-b\}-\frac{w_{0}}{\sqrt{N_{0}}}\sqrt{N_{0}}\{F_{N_{0}}\left( b \right)-b\}$

and therefore

${\tilde{er}_{up}\left( b \right)\approx}_{D}w_{0}+{b(w}_{1}-w_{0})+\frac{w_{1}}{\sqrt{N_{1}}}B_{1}-\frac{w_{0}}{\sqrt{N_{0}}}B_{0}$

where $B_{0}$ and $B_{1}$ are two independent standard BB’s and $\approx_{D}$ denotes approximation in distribution.

Since a linear combination of independent BBs is again a BB, the difference of the last two terms in the above equation can also be approximated by another BB, denoted by $aB(b)$ with $B(b)$ a standard BB and $a=\sqrt{\frac{w_{0}^{2}}{N_{0}}+\frac{w_{1}^{2}}{N_{1}}}$. The above equation can then be restated as:

$\tilde{er}_{up}\left( b \right) \approx_{D}w_{0}+{b(w}_{1}-w_{0})+aB(b)$.

Consequently, under the null hypothesis, the distribution of $\hat{er}_{up}^{*}={min}_{d}\left\{ \tilde{er}_{up}\left( d \right) \right\}$ can be approximated by:

$\hat{er}_{up}^{*}\approx_{D}{w_{0}+min}_{b}\left\{ {b(w}_{1}-w_{0})+aB(b) \right\}$.

The stochastic process $\tilde{er}_{up}\left( b \right)$ is a Brownian bridge **with drift** ${b(w}_{1}-w_{0})$ and to the best of our knowledge an explicit expression for the CDF of its minimum, i.e. the CDF of $\hat{er}_{up}^{*}$, is not known.

However, it is easy to calculate it by simulation using the following steps:

1. Generate $B(b)$ over a fine grid of $d$ values;
2. Find the minimum value of $\left\{ {b(w}_{1}-w_{0})+aB(b) \right\}$ over the grid.
3. Repeat these two steps $M$ times to build up a file of $iid$ copies of the minimum values
4. Calculate the corresponding CDF, providing an asymptotic approximation of the null distribution.
5. Observed values of $\hat{er}_{up}^{*}$ or $\hat{er}_{down}^{*}$can again be compared to the file entries to get asymptotic approximations of their $p$-values.

Assuming the costs of misclassifying subjects are equal for each group, i.e. $w_{0}=w_{1}=\frac{1}{2}$, there is no longer a drift present. Now the associated asymptotic $p$-values can be calculated without simulation since the relevant distribution is known. In this case:

$\hat{er}_{up}^{*}\approx_{D}\frac{1}{2}+{\sqrt{N}{min}_{b}\{B\left( b \right)\}}/{2\sqrt{N_{0}N_{1}}}$

and therefore

$P(\hat{er}_{up}^{*}\leq x)\approx P({min}_{b}\left\{ B(b) \right\}\leq{2\sqrt{N_{0}N_{1}}\left( x-\frac{1}{2} \right)}/\sqrt{N})$.

Now, the CDF of ${min}_{d}\{B\left( b \right)\}$ is given by the simple formula

$P\left( {min}_{d}\left\{ B(b) \right\}\leq t \right)=exp(-2t^{2})$ for $t\leq0$,

refer to [1,2], so that we end up with the simple approximation:

$$P(\hat{er}_{up}^{*}\leq x)\approx exp\{-8\frac{N_{0}N_{1}}{N}\left( x-\frac{1}{2} \right)^{2}\}$$

It is evident from the expression for $\hat{er}_{up}^{*}$ that the numbers $N_{0}$ and $N_{1}$ and the weights $w_{0}$ and $w_{1}$ act on the null distribution in large samples through the two quantities: ${(w}_{1}-w_{0})$ and $a=\sqrt{{w_{0}^{2}}/{N_{0}+{w_{1}^{2}}/{N_{1}}}}$. In particular, if $N_{0}$ and $N_{1}$ are both large while $w_{0}$ and $w_{1}$ differ substantially, then the stochastic part $aB(b)$ has little influence since the factor $a$ is small.

This simulation approximation has the advantage that its computation time does not increase for increasing sample sizes. However, this comes at the cost of two approximations being involved, namely the simulation approximation as well as the large sample approximation.

Assuming equal weights simplifies the approach even further. To see this, substitute $w_{0}=w_{1}=\frac{1}{2}$ into (5) and (6) and write the sums in terms of empirical CDFs $F_{N_{0}}\left( c \right)$ and $F_{N_{1}}\left( c \right)$ to get:

$\hat{er}_{up}\left( c \right)=\frac{1}{2}+\frac{1}{2}[F_{N_{1}}\left( c \right)-F_{N_{0}}(c)]$ and

$\hat{er}_{down}\left( c \right)=\frac{1}{2}+\frac{1}{2}[F_{N_{0}}\left( c \right)-F_{N_{1}}(c)]$.

Consequently:

$$\hat{er}_{up}^{*} =\frac{1}{2}+\frac{1}{2}{{min}_{c}[F}_{N_{1}}\left( c \right)-F_{N_{0}}(c)] =\frac{1}{2}-\frac{1}{2}{max}_{c}[F_{N_{0}}\left( c \right)-F_{N_{1}}(c)]=\frac{1}{2}(1-D^{+})$$

$$\hat{er}_{down}^{*}=\frac{1}{2}+\frac{1}{2}{{min}_{c}[F}_{N_{0}}\left( c \right)-F_{N_{1}}(c)] =\frac{1}{2}-\frac{1}{2}{max}_{c}[F_{N_{1}}\left( c \right)-F_{N_{0}}(c)] =\frac{1}{2}(1-D^{-})$$

where $D^{+}$ and $D^{-}$ are the right and left one-sided two-sample Kolmogorov-Smirnov (K-S) test statistics for comparing the experimental with the control group [1].

The exact null distributions of the K-S statistics are available in standard statistical packages such as SAS [3] from which the relevant $p$-values may be found. Note that when $D^{+}$ and $D^{-}$ are multiplied by the factor $\sqrt{{N_{0}N_{1}}/{(N_{0}+N_{1})}}$ they have the asymptotic CDF $H\left( x \right)=1-\exp\left( -2x^{2} \right), x\geq0$, from which the asymptotic formula for the $p$-values again follows.

**Section S4:**

**Comparing null distributions**

In this section we graphically compare the three approaches to calculating the null CDF, i.e. FS, LOO and Asymptotic approach. To gain some insight into how each approach differs given various combinations of group sizes, weight selections and shift assumptions, we plot $\hat{er}_{up}^{*}$ and $\hat{er}_{min}^{*}$, as well as the asymptotic approximation of the null distribution for the nine scenarios outlined in Table S1. Note that these scenarios are used throughout this supplementary to illustrate findings described in the paper.

| \| **Scenario** \| \| **Control Group** \| \| **Experimental Group** \| \| \| --- \| --- \| --- \| --- \| --- \| --- \| \| **Group Size** \| **Weight** \| **Group Size** \| **Weight** \| \| 1. $w_{0}=w_{1}$ \| (a) $N_{0}=N_{1}$ \| 20 \| $\frac{1}{2}$ \| 20 \| $\frac{1}{2}$ \| \| (b) $N_{0}<N_{1}$ \| 10 \| $\frac{1}{2}$ \| 20 \| $\frac{1}{2}$ \| \| (c) $N_{0}>N_{1}$ \| 20 \| $\frac{1}{2}$ \| 10 \| $\frac{1}{2}$ \| \| 1. $w_{0}>w_{1}$ \| (a) $N_{0}=N_{1}$ \| 20 \| $\frac{3}{4}$ \| 20 \| $\frac{1}{4}$ \| \| (b) $N_{0}<N_{1}$ \| 10 \| $\frac{3}{4}$ \| 20 \| $\frac{1}{4}$ \| \| (c) $N_{0}>N_{1}$ \| 20 \| $\frac{3}{4}$ \| 10 \| $\frac{1}{4}$ \| \| 1. $w_{0}<w_{1}$ \| (a) $N_{0}=N_{1}$ \| 20 \| $\frac{1}{4}$ \| 20 \| $\frac{3}{4}$ \| \| (b) $N_{0}<N_{1}$ \| 10 \| $\frac{1}{4}$ \| 20 \| $\frac{3}{4}$ \| \| (c) $N_{0}>N_{1}$ \| 20 \| $\frac{1}{4}$ \| 10 \| $\frac{3}{4}$ \| |
| --- | --- | --- | --- | --- | --- | --- | --- | --- | --- | --- | --- | --- | --- | --- | --- | --- | --- | --- | --- | --- | --- | --- | --- | --- | --- | --- | --- | --- | --- | --- | --- | --- | --- | --- | --- | --- | --- | --- | --- | --- | --- | --- | --- | --- | --- | --- | --- | --- | --- | --- | --- | --- | --- | --- | --- | --- | --- | --- |
| **Table S1. List of Scenarios.**  A List of the different scenarios used in addition to those reported in the main paper |

Figure S1 shows how the null CDFs of the error rates starts to turn sharply as the error rate increases to the smaller of the two weights. It is therefore evident that the shape of the null distribution depends on the selected weights as well as the group sizes. This proves that the ERp approach accounts for group sizes and selected weight sets. As discussed in the main paper, the discriminatory significance of a variable becomes more apparent when its error rate is evaluated based on its corresponding $p$-value.

|  | $\boldsymbol{N}_{\boldsymbol{0}}\boldsymbol{=}\boldsymbol{N}_{\boldsymbol{1}}$ | $\boldsymbol{N}_{\boldsymbol{0}}\boldsymbol{<}\boldsymbol{N}_{\boldsymbol{1}}$ | $\boldsymbol{N}_{\boldsymbol{0}}\boldsymbol{>}\boldsymbol{N}_{\boldsymbol{1}}$ |
| --- | --- | --- | --- |
| $\boldsymbol{w}_{\boldsymbol{0}}\boldsymbol{=}\boldsymbol{w}_{\boldsymbol{1}}$ | 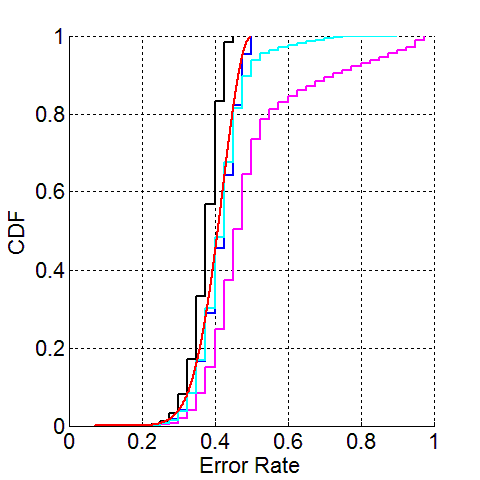 | 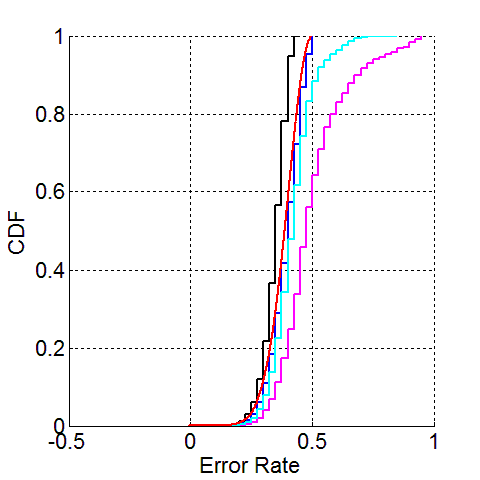 | 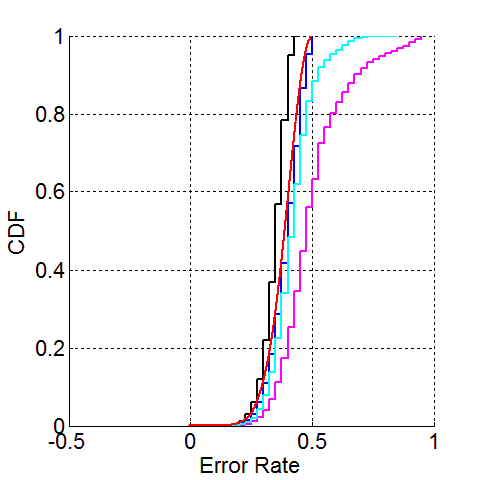 |
| $\boldsymbol{w}_{\boldsymbol{0}}\boldsymbol{>}\boldsymbol{w}_{\boldsymbol{1}}$ | 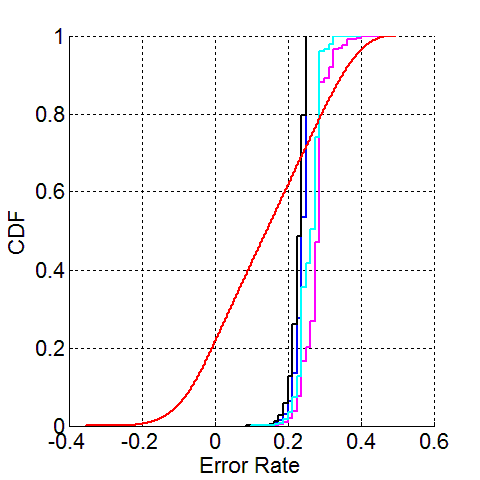 | 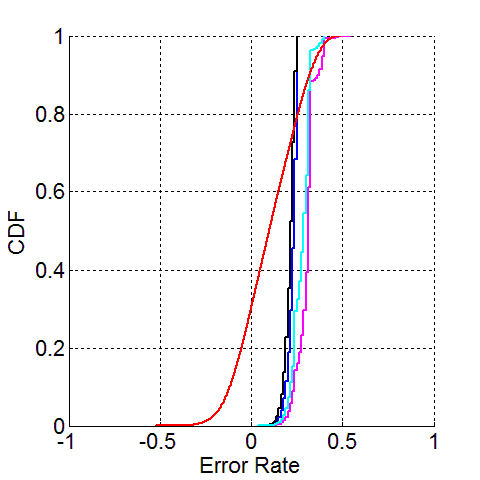 | 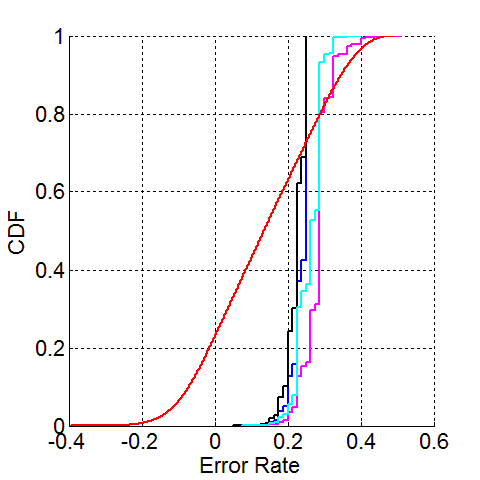 |
| $\boldsymbol{w}_{\boldsymbol{0}}\boldsymbol{<}\boldsymbol{w}_{\boldsymbol{1}}$ | 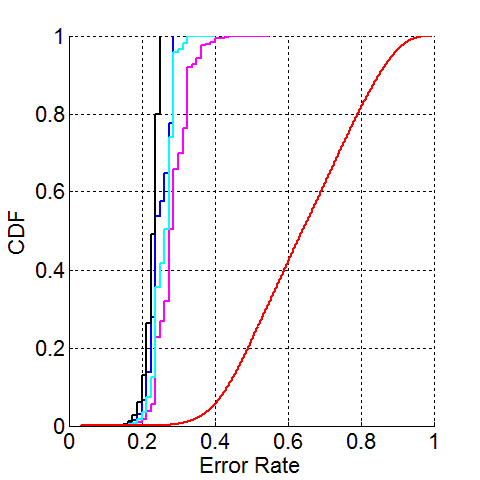 | 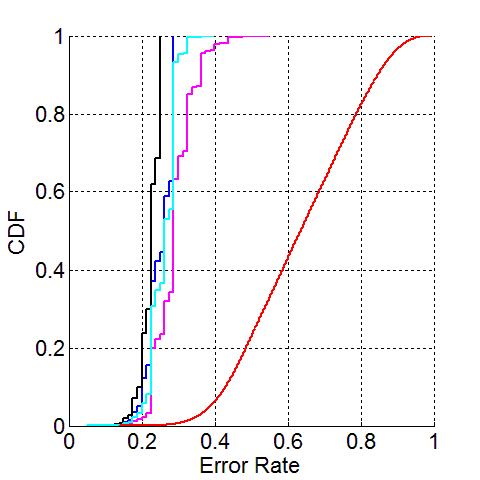 | 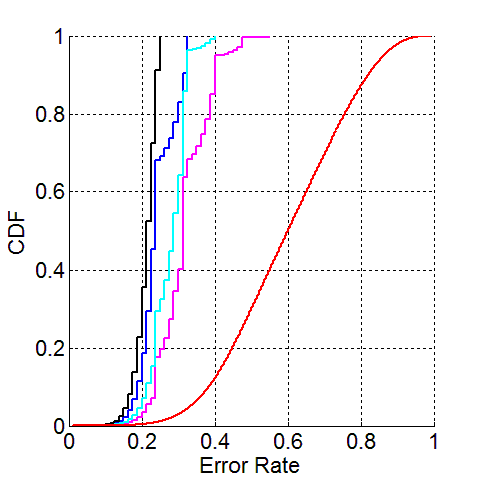 |
| **Figure S1 A graphically comparison of the three approaches to calculating the null CDF.**  Each image in Figure S1 represents a scenario from Table S1. The blue and black lines represent the null CDF’s for $\hat{er}_{up}^{*}$ and $\hat{er}_{min}^{*}$, respectively. The red lines represent the asymptotic approximation of the null CDF. | | | |

**Section S5:**

**Tests based on leave-one-out error rates**

Consider first the amendments required for the error rate of the upward rule. Let $\hat{er}_{up}(c,i)$ denote the error rate in (5), but with the $i^{th}$ subject excluded from the summations on the right hand side of the equation. Also let $\hat{c}_{up}(i)$ denote the threshold level $c$ that minimizes $\hat{er}_{up}(c,i)$ over $c$ and let $\hat{er}_{up}^{*}(i)$ denote the minimized value of $\hat{er}_{up}(c,i)$. Using the upward rule the $i^{th}$ subject is classified as an experimental subject if $x_{i}>\hat{c}_{up}(i)$ and as a control subject if $x_{i}\leq\hat{c}_{up}(i)$. Weighting and summing over all the excluded cases, the LOO error rate for the upward rule can be expressed as

| $\hat{el}_{up}=\frac{w_{0}}{N_{0}}\sum_{i=1}^{N} (1-y_{i})I(x_{i}>\hat{c}_{up}(i))+\frac{w_{1}}{N_{1}}\sum_{i=1}^{N} y_{i}I(x_{i}\leq\hat{c}_{up}(i))$ |  |
| --- | --- |

One generally finds that $\hat{el}_{up}$ is somewhat larger than $\hat{er}_{up}^{*}$ which is to be anticipated since $x_{i}$ is not able to influence the choice of $\hat{c}_{up}(i)$and this leads to reduction in bias of $\hat{el}_{up}$ as an estimator of ${er}_{up}^{*}$ as compared to $\hat{er}_{up}^{*}$. For the downward shift rule $\hat{el}_{down}$ is defined analogously. There is more than one way to define the minimum LOO error rate. However, we restrict our discussion to the simplest choice, namely to take $\hat{el}_{min} =min\{\hat{el}_{up},\hat{el}_{down}\}$ and the associated direction as $\hat{dl}_{min} ="up"$ if $\hat{el}_{up}<\hat{el}_{down}$ and $\hat{dl}_{min}="down"$ otherwise.

The test statistics derived above are all non-parametric, with $\hat{el}_{up}$ and $\hat{el}_{down}$ having the same distribution. To see this, consider first $\hat{el}_{up}$ and put $u_{n}=F(x_{n})$ and $b=F(c)$ as before, so that $\hat{el}_{up}$ can be expressed as

| $\hat{el}_{up}=\frac{w_{0}}{N_{0}}\sum_{i=1}^{N} (1-y_{i})I(u_{i}>F(\hat{c}_{up}\left( i \right)))+\frac{w_{1}}{N_{1}}\sum_{i=1}^{N} y_{i}I(u_{i}\leq F(\hat{c}_{up}\left( i \right)))$ |  |
| --- | --- |

Suppose firstly that $y_{i}=0$, i.e. subject $i$ is in the control group. Then

| $\hat{er}_{up}(c,i) =\frac{w_{0}}{N_{0}-1}\sum_{n=1,n\neq i}^{N} (1-y_{n})I(x_{n}>c)+\frac{w_{1}}{N_{1}}\sum_{n=1}^{N} y_{n}I\left( x_{n}\leq c \right)$ |  |
| --- | --- |
| $=\frac{w_{0}}{N_{0}-1}\sum_{n=1,n\neq i}^{N} (1-y_{n})I(u_{n}>b)+\frac{w_{1}}{N_{1}}\sum_{n=1}^{N} y_{n}I\left( u_{n}\leq b \right)$ |  |

Minimising over $c$ is equivalent to minimising over $b=F(c)$ so that $F(\hat{c}_{up}\left( i \right))=\hat{b}_{up}\left( i \right)$ which minimises $\hat{er}_{up}(c,i)$ and is therefore only a function of the $u_{n}$’s (other than the $u_{i}$’s). Similarly for the case $y_{i}=1$. Hence $\hat{el}_{up}$ can be restated as

| $\hat{el}_{up}=\frac{w_{0}}{N_{0}}\sum_{i=1}^{N} (1-y_{i})I(u_{i}>\hat{b}_{up}(i))+\frac{w_{1}}{N_{1}}\sum_{n=1}^{N} y_{i}I(u_{i}\leq\hat{b}_{up}\left( i \right))$ |  |
| --- | --- |

which shows that $\hat{el}_{up}$ is also only a function of the $u_{n}'$s and its null distribution therefore does not depend on the true common CDF $F$ under the null hypothesis. A similar argument holds for $\hat{el}_{down}$. It can also be seen that the expressions involved in the upward and downward cases differ only on sets carrying zero probability under the null hypothesis assumption. Finally, $\hat{el}_{min}$ can also be expressed as a function of the $u_{n}$’s and is therefore also a non-parametric test statistic.

**Section S6:**

**Power comparisons of test statistics**

This section compares the power of the following four error rate estimators or test statistics: $\hat{er}_{up}^{*}$; $\hat{er}_{min}^{*}$; $\hat{el}_{up}$ and $\hat{el}_{min}$. That is, when an upward shift is assumed and explicitly tested ($\hat{er}_{up}^{*}$ and $\hat{el}_{up}$), as well as when no assumption is made and both shift directions are evaluated ( $\hat{er}_{min}^{*}$ and $\hat{el}_{min}$). Furthermore, we also compare our four error rate estimators or test statistics to the Mann-Whitney (MW) test statistic as it represent a standard non-parametric two-sample test which is known to be powerful.

The scenarios listed in Table S1 (with $w_{0}<w_{1}$)were evaluated under three different distributional assumptions to incorporate some of the known characteristics of metabolomics data: (***Scenario 1****)* Values for control subjects followed a $N(0,1)$ distribution while those of experimental subject where drawn from a $N(\mu,1)$ distribution where $\mu$ assumed different values; (***Scenario 2****)* Values for control subjects followed a $LN(0,1)$ distribution while those of experimental subject where drawn from a $LN(\mu,1)$ distribution where $\mu$ assumed different values; and (***Scenario 3****)* Values for control subjects followed a $\Gamma(0,1,1)$ distribution while those of experimental subject where drawn from a $\Gamma\left( 0,\beta,1 \right)$ distribution where $\beta$ (the scale parameter) assumed different values.

As a first comparison, the resulting $p$-values were averaged over ten thousand repetitions to measure the expected power of the test statistics. As a second measure, we calculated the proportion of the ten thousand $p$-values resulting from each simulation for the test statistics $\hat{er}_{min}^{*}$, $\hat{el}_{up}$, $\hat{el}_{min}$ and MW which turned out to be lower than those of $\hat{er}_{up}^{*}$, at each shift magnitude. These proportions were evaluated against a 50% threshold which if exceeded indicates that this particular test statistic out performs $\hat{er}_{up}^{*}$.

We found that $\hat{er}_{up}^{*}$ is on average the most powerful of the four error rate estimates. Furthermore, $\hat{er}_{up}^{*}$ also has a high probability of being more powerful than $\hat{el}_{up}$,$\hat{er}_{min}^{*}$, $\hat{el}_{min}$ and MW for larger shifts. The MW is the only test statistic that outperforms $\hat{er}_{up}^{*}$ but only for smaller shifts, below 1.2 given a normal or log-normal distribution and below 1 given a gamma distribution. However, we have shown in the main paper that shifts of these magnitudes are not significant. Only for the scenario where $w_{0}<>w_{1}$ do we find that the $\hat{er}_{up}^{*}$ test statistic only outperforms the MW for larger shifts in distribution. However, it is important to note that the MW test cannot account for the weight selection which is no longer equal and cannot classify new subjects or account for unequal cost of misclassification into the two groups. Figure S2 is an excerpt from these results.

|  |  | $\boldsymbol{N}_{\boldsymbol{0}}\boldsymbol{<}\boldsymbol{N}_{\boldsymbol{1}}$ | $\boldsymbol{N}_{\boldsymbol{0}}\boldsymbol{>}\boldsymbol{N}_{\boldsymbol{1}}$ |
| --- | --- | --- | --- |
| ***Log-Normal*** | $\boldsymbol{w}_{\boldsymbol{0}}\boldsymbol{=}\boldsymbol{w}_{\boldsymbol{1}}$ | 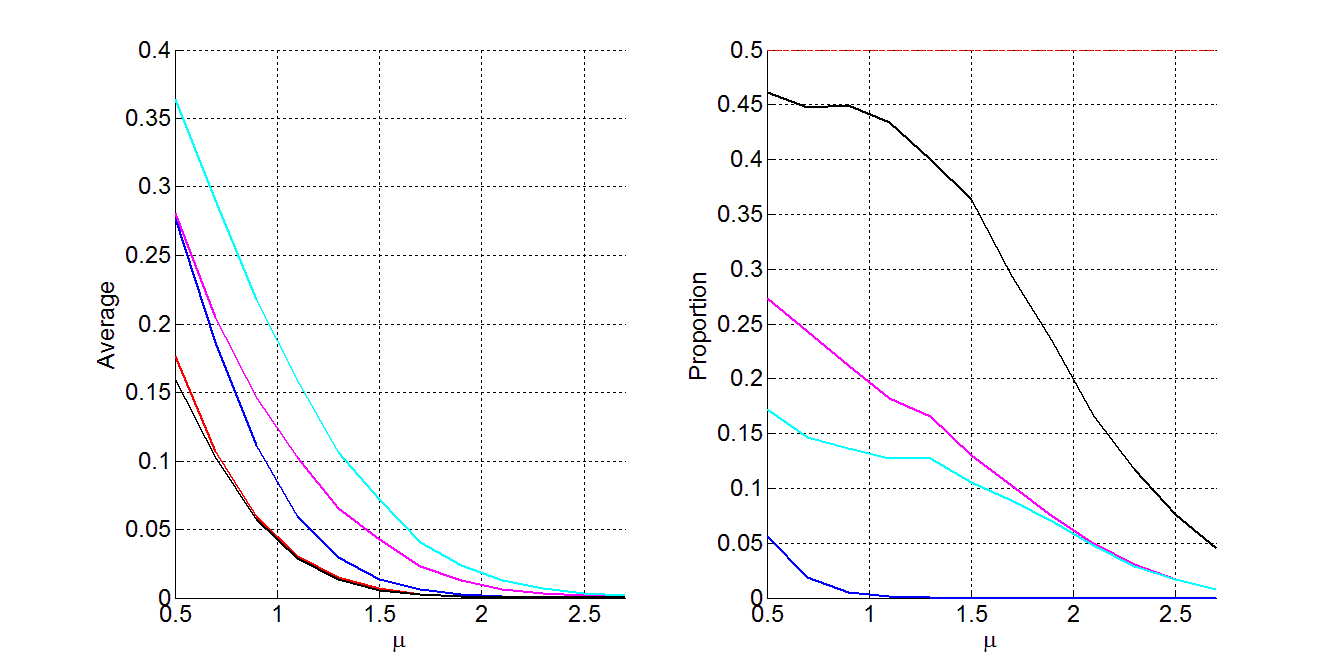 | 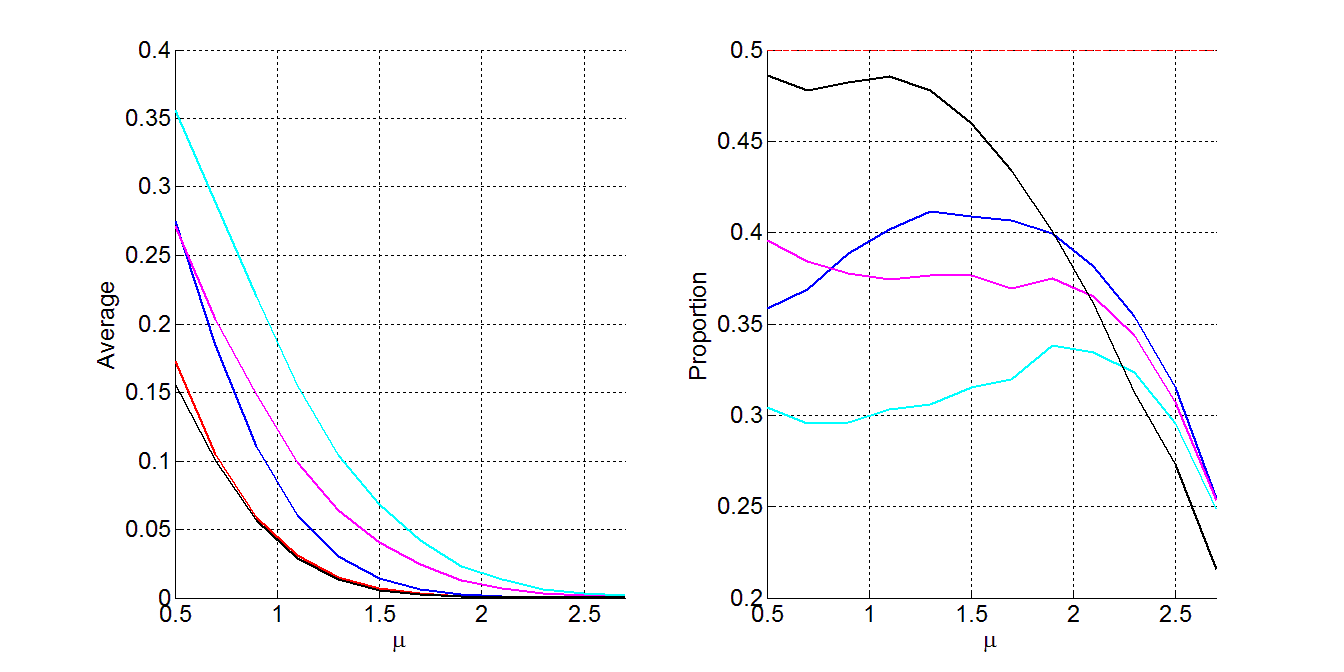 |
|  | $\boldsymbol{w}_{\boldsymbol{0}}\boldsymbol{<}\boldsymbol{w}_{\boldsymbol{1}}$ | 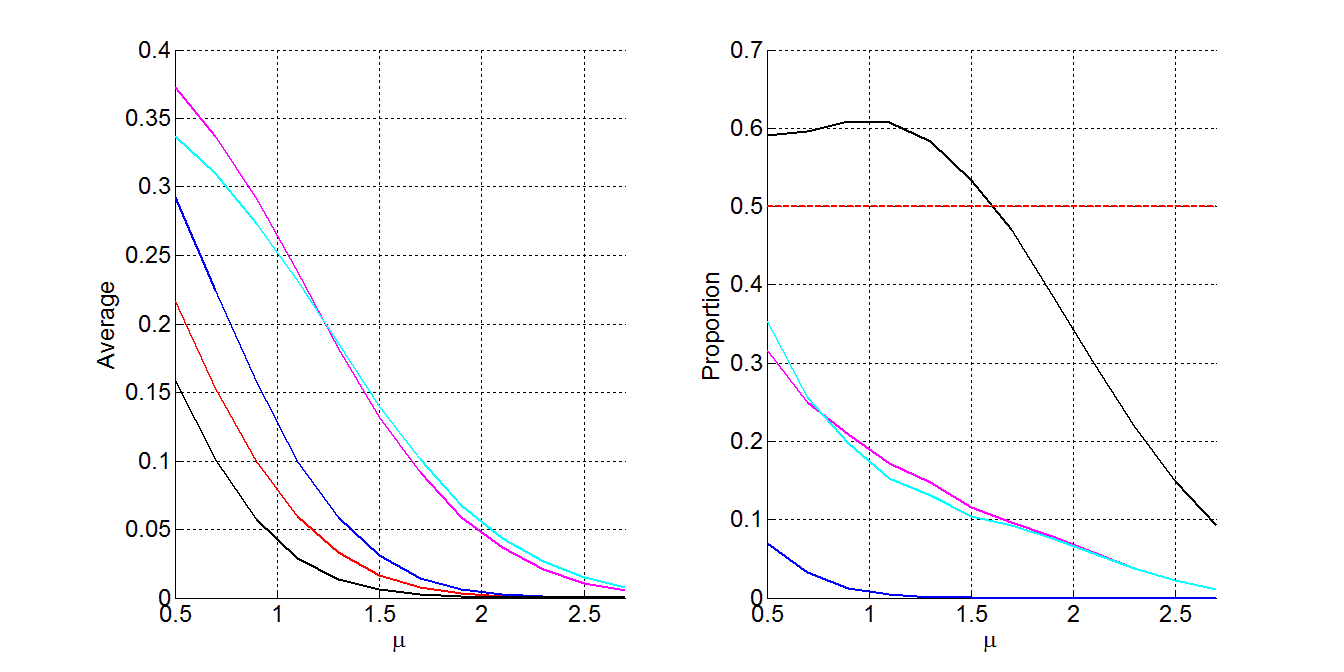 | 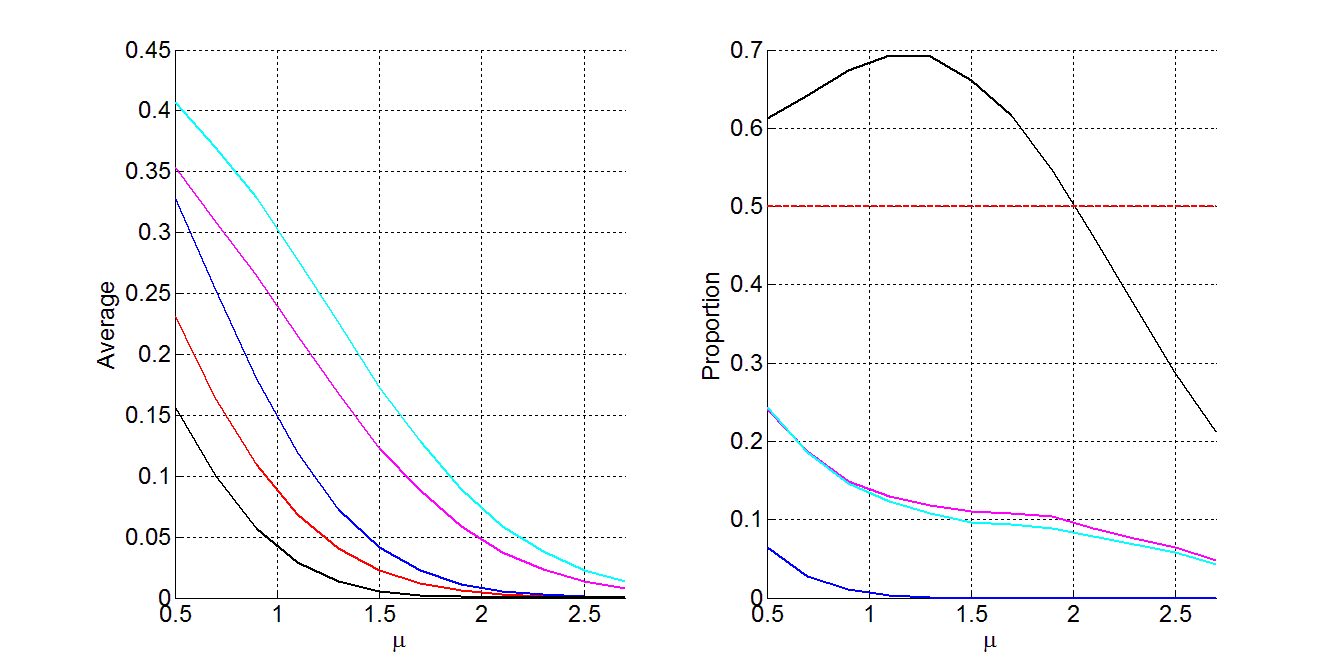 |
| ***Gamma*** | $\boldsymbol{w}_{\boldsymbol{0}}\boldsymbol{=}\boldsymbol{w}_{\boldsymbol{1}}$ | 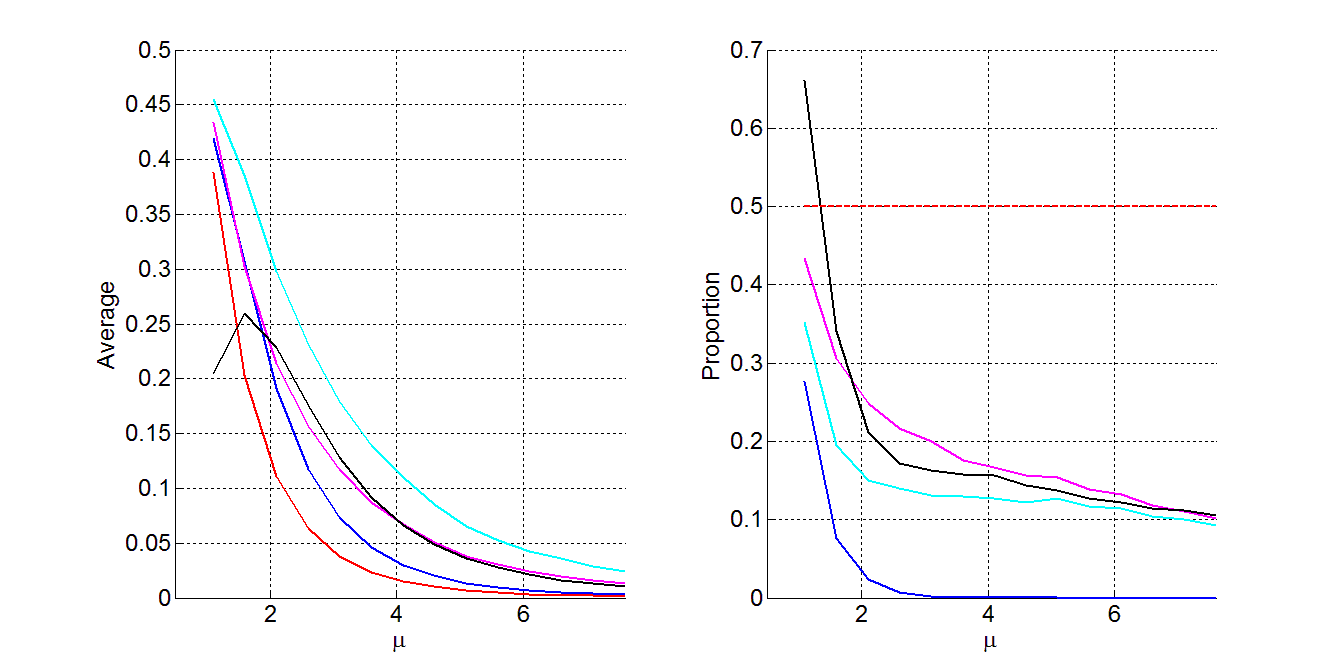 | 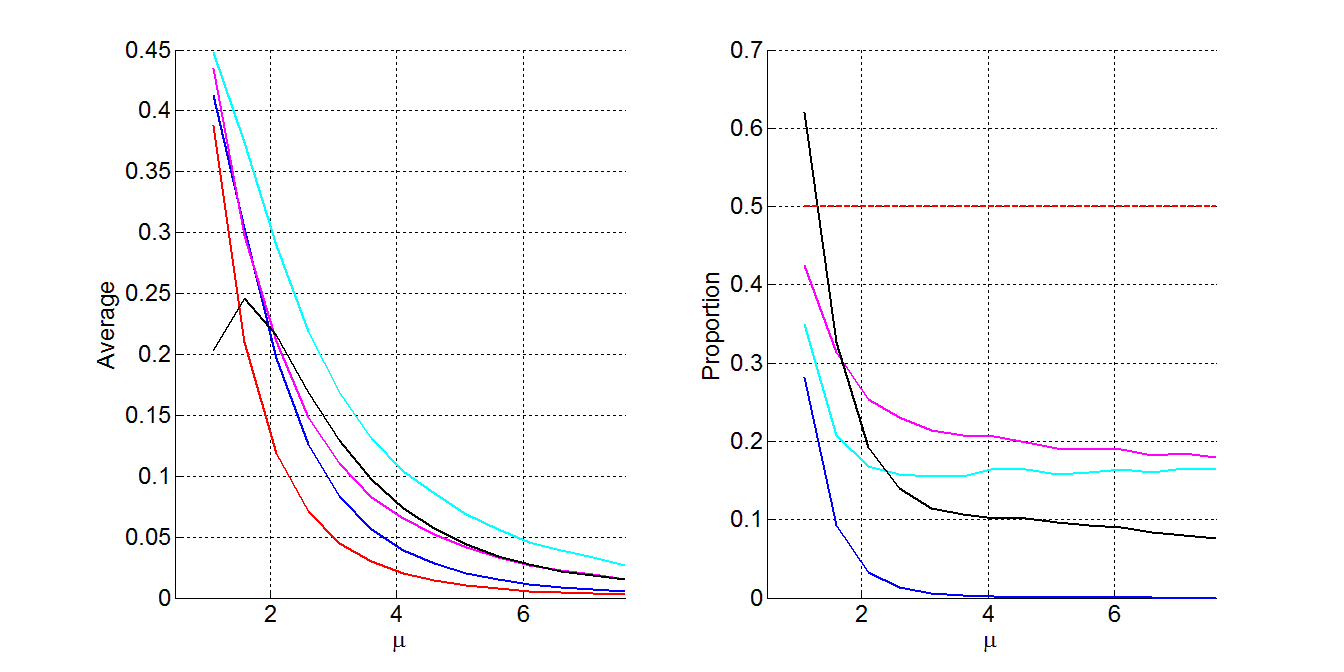 |
|  | $\boldsymbol{w}_{\boldsymbol{0}}\boldsymbol{<}\boldsymbol{w}_{\boldsymbol{1}}$ | 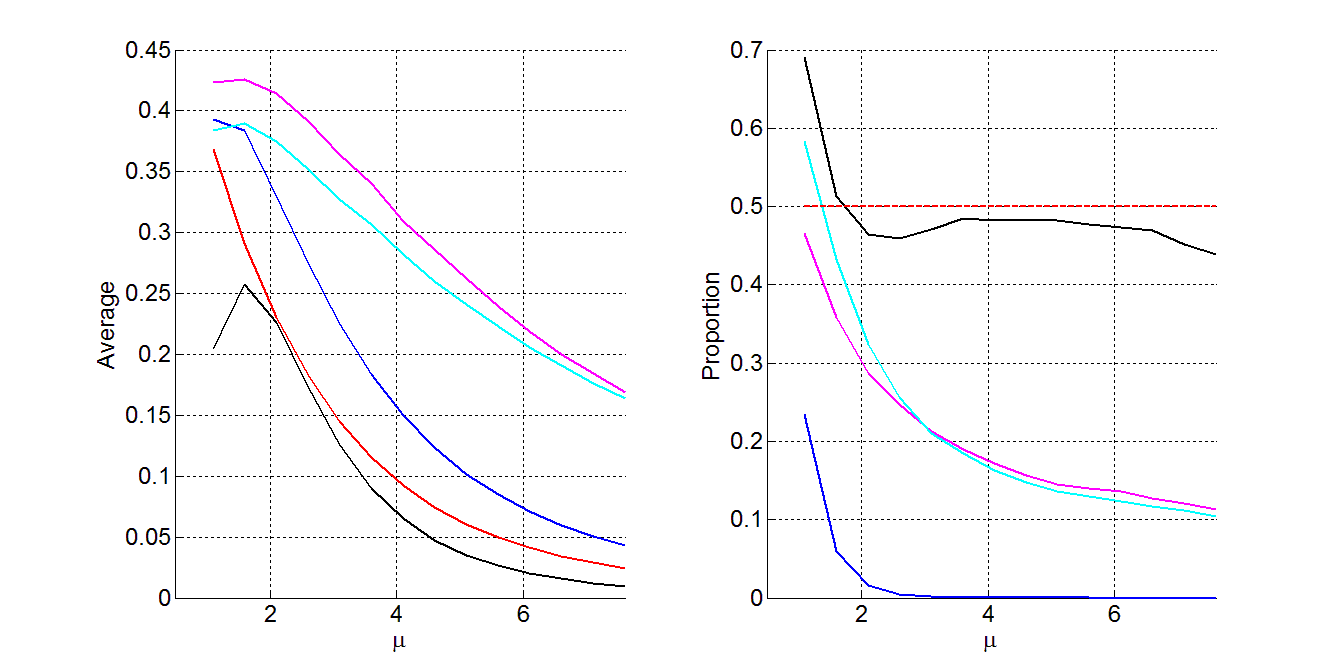 | 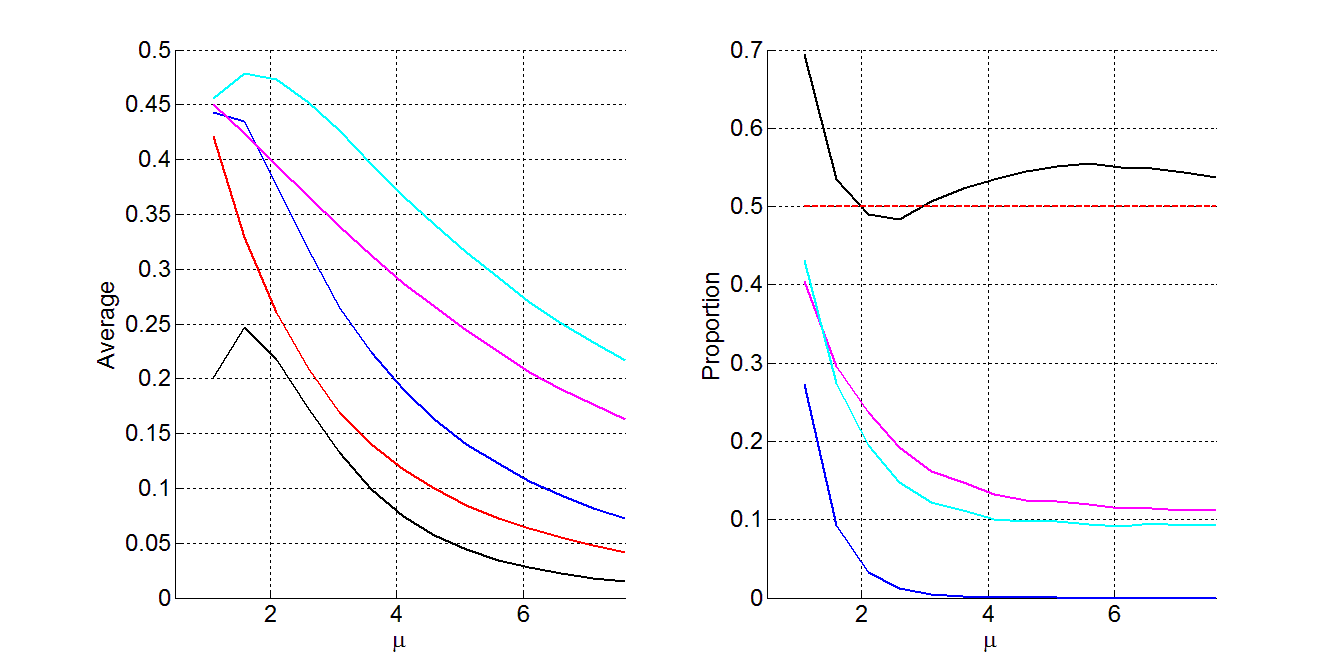 |
| **Figure S2 Power comparison between test statistics assuming a Log-normal distribution**  The left panel of each figure shows the average $p$-values associated with $\hat{er}_{up}^{*}$ (red lines). The right panel shows the proportion below those of $\hat{er}_{up}^{*}$. The dotted red line represents the 50% cut-off. Other statistics are$\hat{er}_{min}^{*}$ (blue lines), $\hat{el}_{up}$ (magenta lines), $\hat{el}_{min}$ (cyan lines) and the MW test statistic (black lines). | | | |

**Section S7:**

**Comparing threshold estimators**

To decide on the relative merits of the three threshold estimates - $\hat{c}_{up}$, $\hat{cl}_{up,1}$ and $\hat{cl}_{up,2}$ - we compared them to the population optimal threshold ($c_{up}$) using a simulation study similar to that reported in the previous section. As described earlier, $c_{up}$ is the choice of $c$ that minimizes ${er}_{up}\left( c \right)$ given by (1). Differentiating (1) and setting the result equal to 0, $c_{up}$ is the solution of the equation

$w_{1}f_{1}\left( c \right)=w_{0}f_{0}\left( c \right)$

where $f_{0}\left( c \right)$ and $f_{1}\left( c \right)$ are the densities (derivatives) of the population CDFs $F_{0}\left( c \right)$ and $F_{1}\left( c \right)$ respectively. Let

$\tau=\frac{w_{1}}{w_{0}}=\frac{f_{0}\left( c \right)}{f_{1}\left( c \right)}$

represent the population threshold can be calculated for each distribution scenario:

*Scenario 1 -* Values for control subjects followed a $N(0,1)$ distribution while those of experimental subject where drawn from a $N(\mu,1)$ distribution where $\mu$ assumed different values

$$f_{0}\left( c \right)=\frac{1}{\sqrt{2\pi}}exp\left( \frac{-c^{2}}{2} \right)$$

$$f_{1}\left( c \right)=\frac{1}{\sqrt{2\pi}}exp\left( \frac{-\left( c-\mu\right)^{2}}{2} \right)$$

$$\tau=\frac{\frac{1}{\sqrt{2\pi}}\exp\left( \frac{-c^{2}}{2} \right)}{\frac{1}{\sqrt{2\pi}}\exp\left( \frac{-\left( c-\mu\right)^{2}}{2} \right)}=exp\left( c\mu-\frac{1}{2}\mu^{2} \right)$$

$\ln\tau= c\mu-\frac{1}{2}\mu$

c$= \left( -\frac{1}{\mu}\ln\tau+\frac{1}{2}\mu\right)$

*Scenario 2 -* Values for control subjects followed a $LN(0,1)$ distribution while those of experimental subject where drawn from a $LN(\mu,1)$ distribution where $\mu$ assumed different values

$f_{0}\left( c \right)=\frac{1}{c\sqrt{2\pi}}exp\left( \frac{-\left( \ln c \right)^{2}}{2} \right)$

$f_{1}\left( c \right)=\frac{1}{c\sqrt{2\pi}}exp\left( \frac{-\left( \ln c-\mu\right)^{2}}{2} \right)$

$$\tau=\frac{\frac{1}{c\sqrt{2\pi}}exp\left( \frac{-\left( \ln c \right)^{2}}{2} \right)}{\frac{1}{c\sqrt{2\pi}}exp\left( \frac{-\left( \ln c-\mu\right)^{2}}{2} \right)}=exp\left( \mu\ln c-\frac{1}{2}\mu^{2} \right)$$

$\ln\tau= \mu\ln c-\frac{1}{2}\mu^{2}$

$$c= exp\left( -\frac{1}{\mu}\ln\tau+\frac{1}{2}\mu\right)$$

*Scenario 3 -* Values for control subjects followed a $\Gamma(0,1,1)$ distribution while those of experimental subject where drawn from a $\Gamma\left( 0,\beta,1 \right)$ distribution where $\beta$ (the scale parameter) assumed different values.

$f_{0}\left( c \right)=exp\left( -c \right)$

$f_{1}\left( c \right)=exp\left( \frac{-c}{b} \right)\frac{1}{b}$

$$\tau=\exp\left( \frac{c}{b}-c \right)b$$

$$\ln\tau=c\left( \frac{1}{b}-1 \right)+\ln b$$

$$c= \frac{\ln\tau-\ln b}{\left( \frac{1}{b}-1 \right)}$$

The bias and mean squared error (MSE) were estimated by averaging the differences and squared differences from $c_{up}$ over the ten thousand estimated values of $\hat{c}_{up}$ , $\hat{cl}_{up,1}$ and $\hat{cl}_{up,2}$ at each shift magnitude ($\mu$). Figures S3A and C (weight set 1) and S3B and D (weight set 2) depict bias and MSE as functions of the shift parameter ($\mu$) respectively. Figures 3A and B both show a positive bias for all estimators. Differences in the bias of the estimators are more severe for smaller shifts, but diminish rapidly when shifts become larger. For shifts exceeding 1.2 (required to get the $p$-values low enough to suggest discriminatory content in the variable) no single estimator distinguishes itself as superior for weight set 1, with $\hat{cl}_{up,2}$ showing the least bias assuming unequal weights. From Figures 3C and D, it is evident that $\hat{cl}_{up,1}$ is preferable as it has a smaller MSE.

| 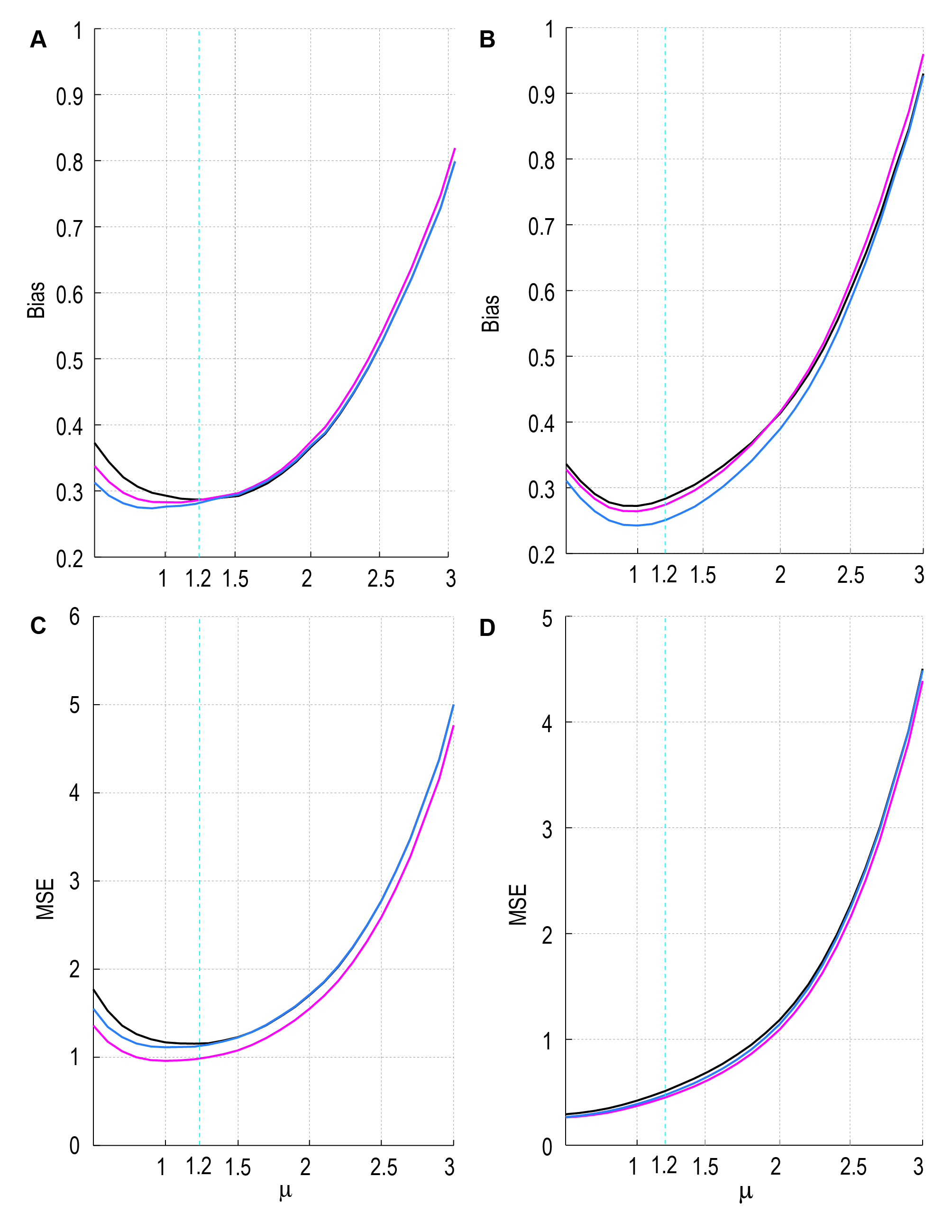 |
| --- |
| **Figure S3**. **Simulation comparison of** t**he threshold estimates.** Observations for the control group were drawn from a $LN(0,1)$ distribution with $N_{0}=21$, while observations for the experimental group were drawn from an upward shifted $LN(\mu,1)$ distribution with $N_{1}=12$. Figure A and B show the bias while Figure C and D show the MSE of $\hat{c}_{up}$ (black lines), $\hat{cl}_{up,1}$ (magenta lines) and $\hat{cl}_{up,2}$ (blue lines) as estimates of the optimal population threshold $c_{up}$. Figures A and C show results for weight set 1 while Figures B and D show results for weight set 2. The dashed blue lines represent points of reference discussed in the text. |

Thresholds were also compared based on the three distributional scenarios discussed in the previous section for the various weight and sample size combinations listed in Table S1. We find that the bias and MSE for $\hat{cl}_{up,1}$ and $\hat{cl}_{up,2}$ are consistently lower than that of $\hat{c}_{up}$, with $\hat{cl}_{up,1}$ being the least bias in most scenarios. The MSE for both LOO estimators are very comparable for small shifts in distribution, however, as shifts become larger, $\hat{cl}_{up,1}$ outperforms $\hat{cl}_{up,2}$ in the majority of scenarios. In fact $\hat{cl}_{up,2}$ only has lower MSE values when group sizes are equal.

**Section S8:**

**Summary of the ERp Approach**

Here we describe the ERp approach as a variable selection and binary classification method. The method was programmed separately in SAS [3] and Matlab [4] and their outputs were compared to ensure the accuracy and reliability of the reported results. The Matlab program is provided in addition to this document.

As depicted in Figure S4, the following inputs are required: (i) A training data matrix **X** the rows of which provide the measurements for each subject and columns contain the observed values of any number of non-categorical variables as potential discriminators. (ii) The variable names in a separate vector $V$. (iii) A categorical vector $Y$ whose entries indicate the group membership of the subjects in each row of **X**, with 0 representing the control group and 1 the experimental group. (iv) The cost of misclassification into one group relative to the other, i.e. the weight pair $w_{0}$ and $w_{1}$ specified, as separate variables, such that the pair sums to 1. (v) A vector $D$ containing the shift direction of interest for each variable (i.e. columns of $\boldsymbol{X}$**)** determining the directional test statistic to be used, with the value 1 referring to “upwards”, -1 to downwards and 0 to no specified direction. Lastly, (vi) the value of the preferred FWER α as a separate variable.

Once these inputs are presented to the program, the number of observations within each group is determined ($N_{0}$ and $N_{1}$) and used along with the weight pair ($w_{0}$ and $w_{1}$) to generate the null distributions required, depending on the shift direction indicated. As mentioned ERp based on the FS error rates is more powerful so that only the FS null distributions are generated. Note that this is done only once regardless of the number of variables since the null distributions depend only $N_{0},$ $N_{1}, w_{0}$ and $w_{1}$. Next the desired FS minimised error rate as well as the corresponding the threshold value is calculated for each variable. Minimised error rates are converted to their corresponding $p$-values by referencing the appropriate null distribution.

Finally, the list of significantly shifted variables is produced with cut-off provided by the BH method to control the FWER at the level α. In the event that a group of variables have the same error rate (and therefore the same $p$-values) but one or more are not significant when the BH rule is applied, then to be on the conservative side regarding control of the FWER, all in the group should be deemed not significant and excluded from the list. This list may then be used for biological interpretation. If variables expected to be significantly shifted, based on subject knowledge, are not found in the list, one may look at the sensitivity of the list when the FWER α is varied. This may be especially relevant when we have small sample sizes and thus insufficient power to detect small shift effects. If the sample size is reasonable, one should investigate alternative reasons before compromising the preferred FWER. After the researcher is comfortable that the results are biologically relevant, clinical consideration can be taken into account to select the final list used to classify new subjects.

| **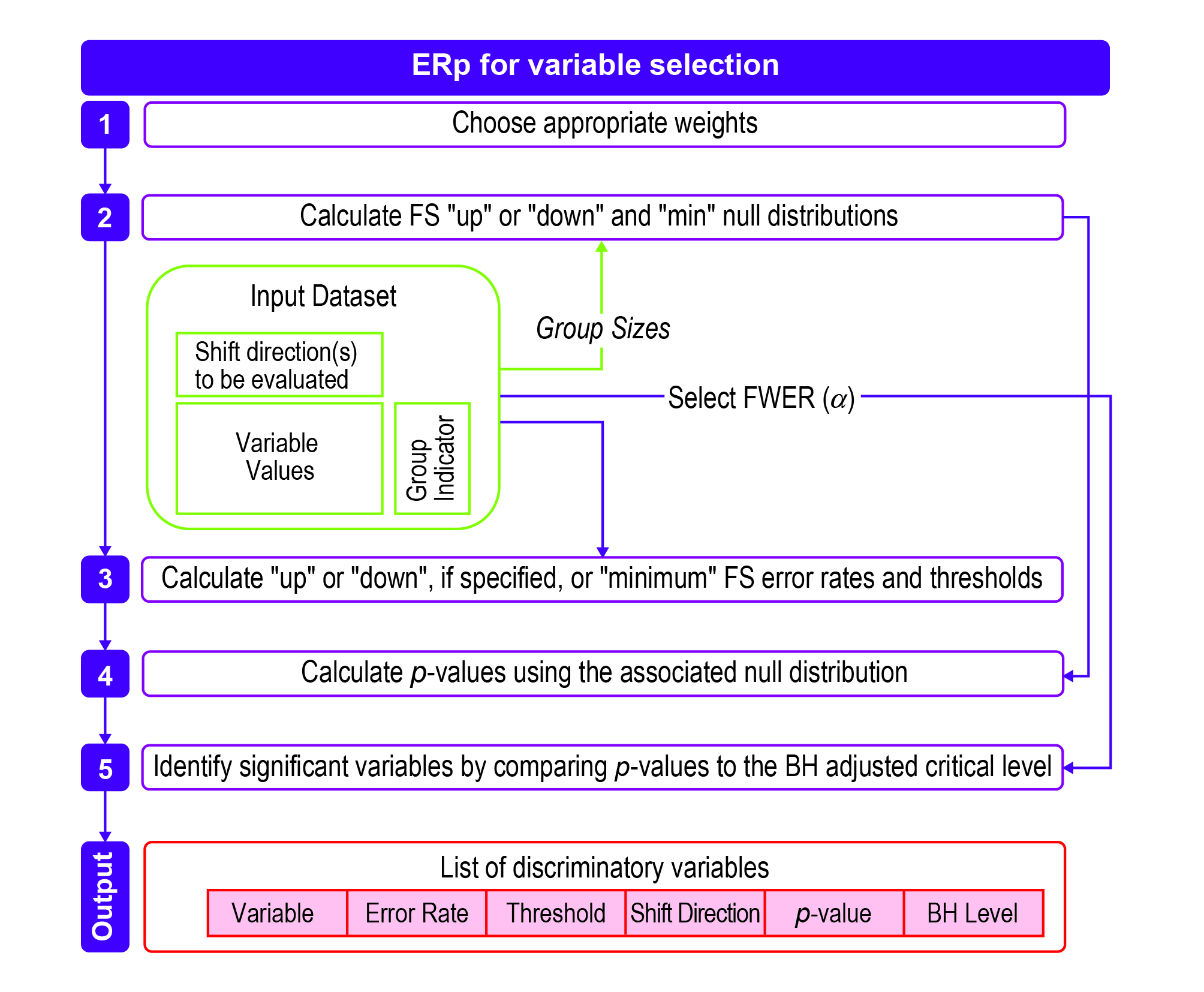** |
| --- |
| **Figure S4**. **The ERp method for variable selection.** The algorithm requires five user inputs: (i) The weight pair $w_{0}$ and $w_{1}$; (ii) The measured variables (i.e. metabolite concentrations) along with their names; (iii) The group indicators coded 0 for the control group and 1 for the experimental group; (iv) The directional indicators (“up” or “down” or not specified) and (v) The family wise error rate α. The algorithm produces a table containing: (a) The list of names of selected variables; (b) The error rate used to test the specified directional hypothesis, i.e. $\hat{er}_{up}^{*}$ if an upward shift was specified; $\hat{er}_{down}^{*}$ if a downward shift was specified; or $\hat{er}_{min}^{*}$ if no shift direction was specified; (c) The threshold associated with each error rate; (d) The shift direction as specified or, if no direction was specified, the direction assigned by ERp (“up” if $\hat{er}_{up}^{*}>\hat{er}_{down}^{*}$ or down if $\hat{er}_{up}^{*}<\hat{er}_{down}^{*}$); (e) The $p$-value associated with the error rate. (f) The BH critical levels for for multiple testing to control the FWER |

Each listed variable can now provide a classification of a new subject using the corresponding threshold value and shift direction (provided or calculated). If the variables are not unanimous in their classification we use a majority vote. However, this implies giving equal credence to all variables in the list, while variables with smaller $p$-values should be considered better discriminators. It may be more reasonable to use a summary classifier that takes this into account. We will not discuss such a “weighted vote” in this paper, but will address it in future research.

**Reference**

[1] Breiman L: **Probability**. Reading, Massachusetts: Addison-Wesley; 1968:282 and 290

[2] Dudley RM: **Uniform Central Limit Theorems**. In *Cambridge Studies in Advanced Mathematics*, 63. Cambridge, UK: Cambridge University Press; 1999:129 and 334-335

[3] SAS Institute Inc. 2011 The SAS System for Windows Release 9.3 TS Level 1M0 Copyright© by SAS Institute Inc., Cary, NC, USA

[4] MATLAB and Statistics Toolbox Release 2012b, The MathWorks, Inc., Natick, Massachusetts, United States.
